# Engineering the maturation of stem cell-derived cardiomyocytes

**DOI:** 10.3389/fbioe.2023.1155052

**Published:** 2023-03-22

**Authors:** Yi Hong, Yun Zhao, Hao Li, Yunshu Yang, Meining Chen, Xi Wang, Mingyao Luo, Kai Wang

**Affiliations:** ^1^ Key Laboratory of Molecular Cardiovascular Science, Department of Physiology and Pathophysiology, School of Basic Medical Sciences, Peking University, Ministry of Education, Beijing, China; ^2^ Center of Vascular Surgery, Fuwai Hospital, National Center for Cardiovascular Diseases, Chinese Academy of Medical Sciences and Peking Union Medical College, Beijing, China; ^3^ Department of Vascular Surgery, Fuwai Yunnan Cardiovascular Hospital, Affiliated Cardiovascular Hospital of Kunming Medical University, Kunming, Yunnan, China; ^4^ State Key Laboratory of Cardiovascular Disease, Fuwai Hospital, Beijing, China

**Keywords:** stem cell, cardiomyocytes, maturation, physical cues, biochemical cues, metabolic engineering, 3D cardiac organoid, *in vivo* implantation

## Abstract

The maturation of human stem cell-derived cardiomyocytes (hSC-CMs) has been a major challenge to further expand the scope of their application. Over the past years, several strategies have been proven to facilitate the structural and functional maturation of hSC-CMs, which include but are not limited to engineering the geometry or stiffness of substrates, providing favorable extracellular matrices, applying mechanical stretch, fluidic or electrical stimulation, co-culturing with niche cells, regulating biochemical cues such as hormones and transcription factors, engineering and redirecting metabolic patterns, developing 3D cardiac constructs such as cardiac organoid or engineered heart tissue, or culturing under *in vivo* implantation. In this review, we summarize these maturation strategies, especially the recent advancements, and discussed their advantages as well as the pressing problems that need to be addressed in future studies.

## 1 Introduction

With the successful isolation and culturing of human embryonic stem cells (hESCs) (Thomson et al., 1998) and the advent of human induced pluripotent stem cells (hiPSCs) (Takahashi et al., 2007), stem cell engineering has stepped into a new era. Stem cell-derived cells, such as pancreatic β cells, endothelial cells (SC-ECs), and cardiomyocytes (SC-CMs), provide unlimited resources for disease modeling and drug discovery. Owing to the essential role of cardiomyocytes (CMs) in maintaining blood pressure and normal physiological activities, several studies have focused on establishing efficient differentiation protocols for generating SC-CMs. SC-CMs are induced by sequential specification on mesoderm, cardiac mesoderm, and cardiac lineages. Currently, the most concise and robust protocol applied a Wnt-on/Wnt-off strategy by using CHIR99021 (GSK-3β inhibitor) and IWP2/IWP4 (Wnt inhibitor) to temporarily modulate canonical Wnt signaling, known as GiWi (GSK-inhibition/Wnt-inhibition), generating up to 98% cTnT^+^ cells on several human pluripotent stem cells lines (Lian et al., 2012). Besides, KDR^+^ PDGFRα^+^ cardiac mesoderm can also be generated with the stimulation of activin and BMP4 under optimized concentration in different hPSC lines (Kattman et al., 2011). Based on genetic modification or the metabolic properties of CMs, cell selection can be further performed to eliminate non-CM lineages (Dubois et al., 2011; Ma et al., 2011; Tohyama et al., 2013).

Although huge advances in differentiation and purification have been achieved over the past 10 years, the maturation of SC-CMs is still a major barrier to downstream applications. For disease modeling, many heart diseases, such as dilated cardiomyopathy, hypertrophic cardiomyopathy, and long QT syndrome are adult-onset. With immaturity, even patient iPSC-derived CMs cannot fully recapitulate the landscape of the disease (Reilly et al., 2022). For potential clinical use in myocardial infarction, due to the expression of HCN4, the spontaneous beats of SC-CMs cause ventricular arrhythmias in non-human primates after transplantation (Liu et al., 2018). Thus, developing new methods for the induction of SC-CM maturation is essential.

**FIGURE 1:**
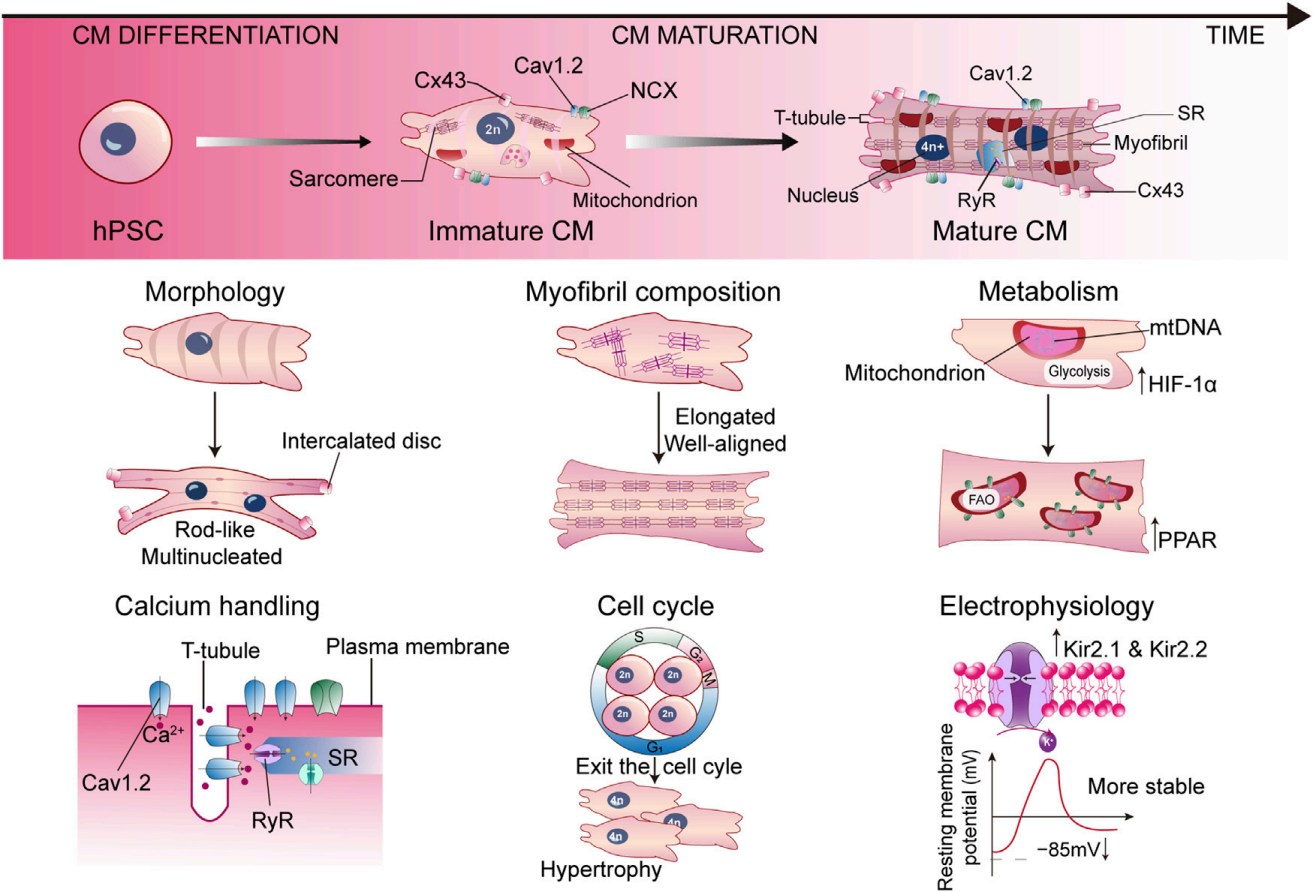
Representative hallmarks for characterizing the mature SC-CM. Cav1.2, L-type Ca^2+^ channel; CM, cardiomyocyte; Cx43, connexin43; HIF-1α, hypoxia inducible factor 1α; hPSC, human pluripotent stem cell; NCX, Na^+^/Ca^2+^ exchanger; PPAR, peroxisome proliferator-activated receptor; RyR, ryanodine receptor; SR, sarcoplasmic reticulum.

Studies on postnatal heart growth in humans and rodents displayed many unique properties of mature CMs in terms of morphology, myofibril composition, sarcomere length, uniaxial beating, electrophysiology, calcium handling, cell cycle, and metabolism, which are absent in SC-CMs (Figure 1). Postnatal CMs exit the cell cycle, therefore heart growth relies on the increasing of CM size and the fusion of adjacent CMs (Li et al., 1996). Along with the fusion, CMs show a large, rod-like, and multinucleated morphology, with the basic structures for electromechanical activities formed. The desmosomes, fascia adherens, and gap junctions are redistributed to the longitudinal ends of CMs to form intercalated discs, joining the cells and supporting the synchronized contraction of the myocardium (Vreeker et al., 2014). The cell membranes invaginate into the center to form T-tubules, delivering Ca^2+^ signaling. The sarcomeres become longer and well aligned, combined with the isoform switch of MYH6 to MYH7, TNNI1 to TNNI3, and TTN-N2BA to TTN-N2B (Guo and Pu, 2020). On the other hand, the expression level of ion channels is dynamically changing during maturation. Downregulation of HCN4 restricts the automatic depolarization to pacemaker cells instead of CMs (Christoffels et al., 2010). An increase of inwardly rectifying potassium channels Kir2.1 and Kir2.2 lowers the resting membrane potential to −85 mV, further stabilizing the membrane (Liu et al., 2010). Proteins associated with Ca^2+^ handling are also upregulated, such as L-type Ca^2+^ channels Cav1.2, ryanodine receptor (RyR), and sarcoplasmic/endoplasmic reticulum calcium ATPase (SERCA), contributing to cardiac dyad formation and appropriate Ca^2+^ flux (Bers, 2002). All these remarkable changes contribute to the functional maturation of CMs. Marked by the aforementioned phenotypes, the maturation of SC-CMs can be evaluated using patch clamp, intracellular imaging, contractile force measurement, RNA-seq, *in vivo* transplantation, or other possible methods.

**FIGURE 2:**
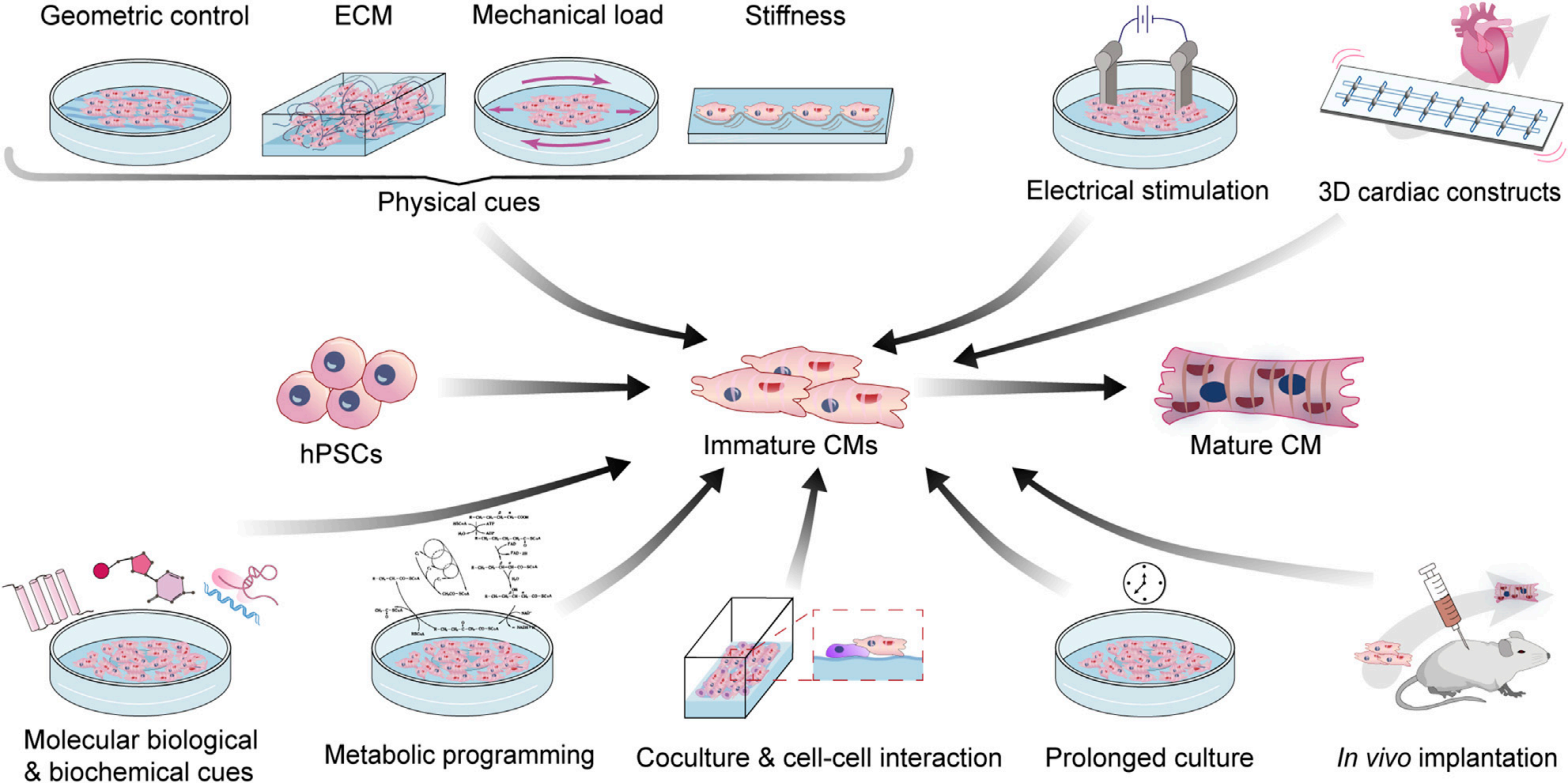
Bioengineering strategies to enhance the maturation of SC-CMs. CM, cardiomyocyte; ECM, extra cellular matrix; hPSC, human pluripotent stem cell.

In this review, we mainly focus on the recent advances in the methodology of SC-CM maturation (Figure 2). We will discuss mechanical, electrical, biochemical, metabolic, and genetic strategies, as well as special platforms such as 3D cardiac tissue constructs and *in vivo* implantation, to engineer the maturation of SC-CMs and conclude with the limitations and future perspectives. The aforementioned major differences between immature and mature CMs, and matching maturation strategies targeting different hallmarks have been summarized in Table 1.

**TABLE 1:** Major differences between immature and mature CMs, and matching maturation strategy.

Hallmarks		Immature CMs (hESC/hiPSC-derived)	Mature CMs	Relevant Maturation Strategy
Structural	Morphology	Small, round shape	Large, rod-like shape, binucleated, elongated	Geometric engineering to provide optimal geometry
				Mechanical load stimulation
	Myofilament	Isotropic, unaligned	Anisotropic, strictly aligned	
	Sarcomere	Unaligned	Assembled and well aligned, elongated	
	Specific structures	Absent	Desmosomes and t-tubule formation	
Functional	Electrophysiology	RP: − 60 ∼ − 70 mV; Shorter APD	RP: ∼ − 85 mV; Longer APD	Electrical stimulation
	Ca^2+^ Handling	Slow, poorly-regulated; Spontaneous Ca^2+^ activity	Rapid, improved handling; Enriched Cav1.2 and RyR	
	Mitochondria	Scattered peripherally or nuclei-adjacent	Higher abundance; Evenly distribution throughout the cell	Metabolism engineering: providing glucose-deprived, FA-rich substrate
				PPAR and AMPK agonists to promote FAO
	Metabolism	Hypoxia; Anaerobic glycolysis predominant	Increased oxygen consumption; Glycolysis-to-FAO shift	
	Contractility	Spontaneous contraction; Low MCF.	Regulated and synchronized contraction; High MCF	Cyclic stretch and progressive stretch
Other	Myocardium stiffness	∼ 6 kPa;	10-40 kPa	Substrate stiffness engineering
	Environmental Cues	*In vitro*; Substrate with relatively simple components	*In vivo*; Complex 3D network of niche cells and ECM	ECM engineering and Co-culture
				3D cardiac tissue construct and *In vivo* implantation

AMPK, adenosine 5'-monophosphate (AMP)-activated protein kinase; APD, active potential duration; CM, cardiomyocyte; dECM, decellularized extracellular matrix; ECM, extracellular matrix; FA, fatty acid; FAO, fatty acid oxidation; hESC, human embryonic stem cell; hiPSC, human induced pluripotent stem cell; MCF, maximum contractile force; PPAR, peroxisome proliferator-activated receptor; RP, resting potential; RyR, ryanodine receptor.

## 2 Mechanical cues

### 2.1 Geometric engineering

One of the most notable distinctions between early SC-CMs and adult CMs is their morphology and microstructure. hSC-CMs cultured on a conventional planar surface exhibit a relatively unspecific round shape, characterized by an isotropic organization of myofilaments and other organelles, and exhibit no sarcolemma invaginations (Gherghiceanu et al., 2011; Aratyn-Schaus et al., 2016; Bedada et al., 2016). In contrast, native adult CMs are more cuboid- or rod-shaped and elongated, with an obvious longitudinal axis and a length-space ratio between 5:1 and 9:1. Additionally, the myofibrils within adult CMs are strictly aligned along the long axis, which is crucial for efficient force production and transduction during contraction (Hampe et al., 2014; Bedada et al., 2016). Another indication of the immaturity of hSC-CMs is the absence of mature sarcomere structures. Moreover, adult CMs are also equipped with a tubular structure known as t-tubules, which extend deep into the myocardium and regulate membrane depolarization as well as contraction synchronization (Ferrantini et al., 2013; Karbassi et al., 2020). These differences in morphology and microstructure have been linked to variations in functionality. Specifically, the aforementioned structural characteristics of adult CMs are associated with enhanced functional properties such as calcium handling, contraction amplitude, contraction force production, and excitation-contraction coupling efficiency. On the other hand, the spontaneous contraction and Ca^2+^ release events indicate the immaturity of SC-CMs (Hampe et al., 2014; Maleckar et al., 2017). Based on these observations, various attempts have been made to reshape and reorganize hSC-CMs to enhance their maturation, both structurally and functionally.

The geometric engineering of the culturing environment has been established as a valid strategy to promote hSC-CM maturation. The most commonly adopted method to engineer the culture geometry is micropatterning of the substrates, which include polydimethylsiloxane (PDMS) (Rao et al., 2013), polylactide-co-glycolide (PLGA) (Khan et al., 2015), Matrigel (Ribeiro et al., 2015), or polyacrylamide (PAA) hydrogel (Strimaityte et al., 2022), using techniques such as electrospinning, photolithography, laser cutting, microcontact, even tissue-specific bio-3D printing (Rao et al., 2013; Salick et al., 2014; Khan et al., 2015; Ribeiro et al., 2015; Rodriguez et al., 2019; Yu et al., 2019; Strimaityte et al., 2022). Recently, another group has developed a matrix mimicking various chemical and physical properties of native heart matrices, including a purposely designed substrate whose ultrastructure resembles the collagen network in natural human hearts (Afzal et al., 2022). These micropatterned substrates generally possess microarchitectures similar to the morphology or organization of mammal (preferably human) adult CMs or decellularized myocardium, serving as scaffolds which guide the geometric growth of hSC-CMs. Newly differentiated hSC-CMs are seeded onto these micropatterned substrates, either in the form of individual CMs (Ribeiro et al., 2015) or aligned cardiac microfibers with cells connected with one another through their distal ends (Rodriguez et al., 2019; Strimaityte et al., 2022). Previous studies have demonstrated that compared to traditional planar substrates, SC-CMs seeded onto geometrically engineered substrates exhibit mature phenotypes similar to adult CMs in terms of morphology (elongated cell shape, improved eccentricity), subcellular organization (anisotropic alignment and development of myofibril, formation of organized sarcomeres and t-tubules, membrane invagination), contractility, calcium handling, and electrophysiological properties (Rao et al., 2013; Salick et al., 2014; Khan et al., 2015; Ribeiro et al., 2015; Rodriguez et al., 2019; Yu et al., 2019; Strimaityte et al., 2022). Interestingly, compared with single CMs, SC-CMs connected end-to-end exhibited faster contraction and relaxation, more mature calcium transients, and a greater proportion of striated t-tubules, highlighting the unique benefits of end-to-end cell connection in the micropatterning method. Additionally, increased expression of gap junction proteins and more synchronized contraction have also been observed in these CMs (Rodriguez et al., 2019; Strimaityte et al., 2022). Geometric engineering through micropatterning is also applicable for generating cardiac organoids or even primitive cardiac chambers, leading to the creation of structurally and functionally robust constructs composed of cardiomyocytes (Ma et al., 2015; Hoang et al., 2018).

While studies have shown that culturing hSC-CMs on substrates with micropatterns that resemble the microstructure of adult CMs can improve maturation, traditional micropatterning methods are typically limited to two-dimensional (2D) substrates. Indeed, 2D systems have unique advantages, such as easy access to studying signaling pathways, metabolic features, and organelle behaviors, and to facilitating high-throughput drug screening (Afzal et al., 2022). However, they cannot adequately represent the native three-dimensional (3D) environment in which human hearts develop. To address this limitation, researchers (Huethorst et al., 2016; Silbernagel et al., 2020) have developed 3D scaffolds with engineered geometric parameters, varying in lengths, widths, and depths, and observed improved maturation of hSC-CMs. Notably, Bertel’s team found that the structural remodeling and reorientation of CMs in these 3D geometric scaffolds led to a higher and more clustered expression of L-type calcium channels and RyRs, as well as the formation of t-tubules, contributing to significantly faster and more robust calcium handling (Silbernagel et al., 2020). This finding shed light on the complex mechanisms through which hSC-CMs respond to geometric cues.

Although a sufficient amount of literature has documented the robust *in vitro* maturation of hSC-CMs through geometric engineering, it remains poorly understood as to how they respond to different geometric cues, and how these cues are associated with their maturation. This lack of knowledge can impede the optimization of geometric parameters used during hSC-CM differentiation and maturation. In addition, the engineering of geometrically designed substrates often relies on the use of certain advanced technologies, such as photolithography, microcontact printing, and laser cutting, which may limit accessibility for non-bioengineering laboratories. Nevertheless, geometric engineering remains a highly promising approach for promoting the maturation of hSC-CMs.

### 2.2 Substrate stiffness

A wide range of studies has established that substrate stiffness serves as an important regulatory factor to the development and maturation of CMs, which is unsurprising given that the stiffness of myocardium does vary across different developmental stages, changing with age. For example, the stiffness of embryonic myocardium is reported to be approximately 6 kPa, while that of healthy murine myocardium is in the range of 10–40 kPa (Ribeiro et al., 2015; Wheelwright et al., 2018). Studies over the years have consistently uncovered the pivotal role of substrate stiffness in promoting the maturation of hSC-CMs through various mechanisms.

Polydimethylsiloxane (PDMS) has been widely employed in manipulating and engineering substrate stiffness, due to its ease of synthesis and potential for modification (Huang and Li, 2011; Arshi et al., 2013; Herron et al., 2018; Rodriguez et al., 2019; Dhahri et al., 2022). Other substances, such as Matrigel (Feaster et al., 2015) and PAA (Ribeiro et al., 2015; Wheelwright et al., 2018) have also been utilized to modulate stiffness. The elastic modulus is typically used to quantify the stiffness of the substrate. A higher elastic modulus indicates a higher level of stiffness and resistance to mechanical force. However, the effects of substrate stiffness on hSC-CMs seem to be inconsistent across studies. Earlier studies suggested that hSC-CMs cultured on PDMS substrates with higher stiffness (99.7 kPa and ∼1,000 kPa, respectively) exhibited improved maturation, particularly in terms of functional maturation (Hazeltine et al., 2012; Arshi et al., 2013). Conversely, more recent literature suggests that softer and more pliable substrates with physiological stiffness ranging from 3.1 kPa to 21 kPa are optimal in terms of increasing myofibril and sarcomere alignment, cell elongation, drug responsiveness, gene expression, and mechanical output, all of which are indicators for CM maturation (Feaster et al., 2015; Ribeiro et al., 2015; Herron et al., 2018; Wheelwright et al., 2018; Rodriguez et al., 2019). In contrast, hSC-CMs cultured on surfaces that are excessively stiff have demonstrated significantly diminished total contraction force, which is associated with the disruption of myofibrils and sarcomere organization (Ribeiro et al., 2015; Rodriguez et al., 2019). A recent study has developed a platform for the large-scale, efficient production of mature hSC-CM based on PDMS-lined roller bottles, which generated greater than 1 × 10^8^ mature CMs per bottle. The CMs matured on this platform also exhibited superior *in vivo* function (Dhahri et al., 2022). Even though the elastic modulus of substrates may vary when different measuring techniques are applied, current evidence seems to suggest that hSC-CMs attain optimal maturation on a relatively soft substrate with physiological stiffness resembling that of healthy, adult myocardium.

The aforementioned studies have led to the discovery of certain mechanisms that account for the interaction between substrate stiffness and hSC-CM maturation. Researchers have established that substrate stiffness can modulate the intracellular tension of shape-engineered CMs, which is a prerequisite for the development of mature myofibrils and, by extension, mature contraction (Ribeiro et al., 2015). Subsequent studies have revealed that stiffness-regulated SC-CM maturation is mediated through the activation of the integrin signaling pathway and focal adhesion kinase, which function as the cellular machinery for force transduction (Herron et al., 2018; Wheelwright et al., 2018).

Despite the advancements that have been made, it is still necessary to form a more comprehensive understanding of the mechanisms by which biophysical cues such as stiffness and shape are translated into cellular and molecular responses, regulating SC-CM maturation. More precise and repeatable elastic modulus measurement is needed to optimize substrate stiffness. The substances currently employed to modulate substrate stiffness are not without limitations. Synthetic materials such as PDMS and PAA are limited in terms of biocompatibility and their tendency to absorb small molecules from the culture, which may impede drug screening for disease models (Rodriguez et al., 2019). On the other hand, biogenic substances such as Matrigel contain undefined ECM proteins, raising questions about the repeatability of results from batch to batch (Herron et al., 2018). In conclusion, substrate stiffness is an essential factor to consider when engineering the optimal culture conditions for hSC-CM maturation.

### 2.3 Extracellular matrix

The extracellular matrix (ECM) provides a highly dynamic and fundamental chemical and physical environment in which cells are directly cultured. Comprised primarily of polysaccharides and collagen proteins secreted by resident cells, the ECM can also encompass a diverse array of other molecules, ranging from small and soluble molecules such as inorganic ions to large and insoluble proteins. These molecules collectively comprise a dynamic microenvironment which plays a crucial role in regulating the overall homeostasis, proliferation, differentiation, development, and maturation of cardiomyocytes (Rienks et al., 2014). As such, the manipulation and optimization of the ECM have been extensively explored as a potential means of enhancing the maturation of hSC-CMs.

#### 2.3.1 Decellularized ECM

Over the past years, several research teams have reported that the cardiac decellularized extracellular matrix (dECM) obtained from adult or fetal bovine cardiac tissue (Fong et al., 2016), pig hearts (Goldfracht et al., 2019; Yu et al., 2019; Mesquita et al., 2021; Xiao et al., 2021), and rat hearts (Hochman-Mendez et al., 2020) can enhance the maturation of SC-CMs, possibly as a result of the specific composition of isogenous tissue, which provides CMs with a relatively favorable and native microenvironment that recapitulates cardiogenesis (Rienks et al., 2014; Sachs et al., 2017; Yu et al., 2019). The dECM can be obtained by chemically removing all matter from the cytoplasm and nucleus using detergents such as sodium dodecyl sulfate (SDS) and Triton X-100 (Xiao et al., 2021). Ideally, the dECM should retain the essential components necessary for CM survival and development, including proteins such as fibronectin, elastin, laminin, fibrillin-1, collagen I, III, IV, and VI (Goldfracht et al., 2019; Hochman-Mendez et al., 2020; Xiao et al., 2021). Some research teams have also explored protocols to decellularize without the use of SDS to minimize disruption to the chemical and structural characteristics of ECM during the process (Goldfracht et al., 2019).

In earlier studies, target hSC-CMs were integrated and repopulated into dECM bioscaffolds with preserved original tissue structure, which confined the dECM to specific sizes and shapes. Subsequently, researchers have exploited the use of soluble cardiac dECM hydrogels (Fong et al., 2016; Goldfracht et al., 2019), which can be engineered into various ideal structures, with optimal mechanical and biochemical properties (Goldfracht et al., 2019). For instance, Yu et al. (2019) developed photocrosslinkable tissue-specific dECM bioinks using dECM hydrogel to 3D bioprint dECM-based tissue constructs, while Goldfracht et al. opted to supplement the dECM hydrogel with genipin and chitosan to achieve desirable mechanical properties (Goldfracht et al., 2019). Mesquita et al. took another path by applying small particles (45–500 μm) derived from pig ventricular dECM to hiPSCs in the early stage of differentiation, also achieving highly effective differentiation of CMs (∼70% of cTnT+) with better maturity in terms of gene expressions, metabolic patterns, electrical behaviors, and responsiveness to dobutamine (Mesquita et al., 2021). Interestingly, one study also reported that human perinatal stem cell-derived ECM (Matrix Plus) can induce hiPSC-CM maturation, with even superior performance compared to Matrigel. This outcome is thought to be related to the elevated level of perlecan present in the Matrix Plus (Block et al., 2020).

By far, pigs have been the most common source for harvesting cardiac dECM, as their cardiac ECM is structurally and biochemically highly similar to that of human hearts, and has yet to exhibit immunogenicity (Efraim et al., 2017). However, the use of xenogeneic ECM that is not yet chemically well-defined may impede the translation to clinical scenarios. Moreover, the current protocols for generating dECM hydrogels often involve the use of certain chemicals, such as polycaprolactone (PCL), polyethylene glycol (PEG), thiolated gelatin, hyaluronic acid (Yu et al., 2019), genipin, and chitosan (Goldfracht et al., 2019). These chemicals serve as either physical support or crosslinker, yet some of which (such as PCL) may be undegradable under physiological conditions, further limiting future bench-to-bedside translation. Furthermore, the precise mechanisms by which tissue-matching dECM promotes CM maturation are not well illustrated, partly owing to the complex nature and composition of dECM (Yu et al., 2019). With that being said, dECM is still an irreplaceable strategy to provide SC-CMs with an *in vitro* complex microenvironment that highly resembles *in vivo* cardiogenesis.

#### 2.3.2 Chemically engineered ECM

ECMs supplemented with defined chemicals, which modulate biochemical, biophysical and other properties of ECM, have been reported to enhance hSC-CM maturation. For instance, Chun et al. found that newly differentiated hiPSC-CMs plated onto combinatorial polymer substrates composed of 4% PEG and 96% PCL (in molar ratio) exhibited significantly improved maturation *via* the integrin-mediated mechanotransductory pathway (Chun et al., 2015). In a subsequent study, Cui et al. (2019) developed a new family of substrates, namely, the monolayer binary colloidal crystals (BCCs), which are crystals composed of two types of different particles with varying material, surface chemistry, and other physical or chemical properties. They found that hiPSC-CMs cultured on 5 μm silica particles and 0.4 μm poly (methyl methacrylate) particles (referred to as 5PM BCCs) showed markedly improved maturation, including improved myofibril ultrastructures and Ca^2+^ handling properties, as well as gene and protein expressions resembling those of adult heart tissue.

The supplementation of defined chemicals, typically polymers, is a straightforward strategy that allows researchers to generate a wide range of substrates with engineered chemical and physical properties tailored to their research focus. Moreover, the chemicals used for engineering the ECM are well-defined, highly commercialized, and user-friendly, with high compatibility with other maturation approaches, making this strategy more applicable for future commercial or clinical applications compared with dECMs.

In summary, the utilization of tissue-specific dECM and chemically engineered ECM have emerged as promising approaches for promoting SC-CM maturation, with potential applications in drug screening and disease modeling. However, the precise underlying mechanisms, as well as the biosafety and biocompatibility of the materials used, remain to be fully elucidated before clinical translation can be realized. Further research may focus on pinpointing the exact key molecules in dECM which are involved in SC-CM maturation as well as the mechanisms they rely on. This can be a giant step towards minimizing undefined, unspecific chemicals and xenogenic materials to further ensure the safety of dECM, paving the way for future clinical translation. In addition, future studies may also assess the potential to utilize optimal ECMs alongside with other strategies such as geometric engineering and electrical stimulation to achieve superb maturation effects.

### 2.4 Mechanical load stimulation

During the process of natural cardiogenesis, CMs are gradually exposed to distinct and specific mechanical loads, including the cyclic contraction force exerted by the surrounding cardiac muscle tissue, as well as the hemodynamic force generated by blood flow. This has led to the hypothesis, and subsequent confirmation, that mechanical load stimulation can enhance the functional and structural maturation of hSC-CMs *in vitro*. The loads utilized to promote *in vitro* SC-CM maturation can be primarily classified into two categories, namely, the stretch load (either static, cyclic, or progressive) and the fluidic load, mimicking the natural loads present during cardiogenesis.

#### 2.4.1 Stretch load stimulation

So far, the most commonly used forms of stretch load are static stretch, cyclic stretch, and progressive stretch. Static stretch entails the provision of continuous and static stress with a fixed strength and direction, thus mechanically elongating the cells to a certain percentage. Cyclic stretch involves cyclically stretching and releasing the CMs at a specific frequency and amplitude, whereas progressive stretch involves gradually increasing the length of the stretched cells over time. These stretch loads are typically applied uniaxially, meaning in only one direction, and require the use of special devices, such as cell stretchers.

Previous studies have primarily applied uniaxial static stress as a form of stretch load. Research has demonstrated that the active twitch force generated by CM contraction increases in proportion to the intensity of the static stress to which CMs are exposed. Turnbull et al. observed that the maximum contractile force (MCF) was positively correlated with the stretch force, reaching 2.3 mN/mm^2^ under the stretch of 20% cell length (Turnbull et al., 2014), while another study reported an MCF of 0.08 mN/mm^2^ after being exposed to 3 weeks of static stretching (Tulloch et al., 2011). The more commonly employed approach is uniaxial cyclic stretching with various frequencies (typically around 1–1.25 Hz), magnitudes (5%–10%), and durations (∼3 days), which better replicates the natural mechanical environment for cardiogenesis. Studies have reported that SC-CMs or SC-CM-based cardiac tissues that underwent cyclic stretching exhibited more mature ultrastructure, such as Z-band and sarcomere alignment, correlated with cardiomyocyte hypertrophy, and higher MCFs (Tulloch et al., 2011; Mihic et al., 2014; Ruan et al., 2015; Shen et al., 2017; Zhang et al., 2017).

Another promising strategy is progressive stretching with increasing length. Kensah’s team applied a growing static stretch (G-stretch) which incrementally elongated the cardiomyocytes by 200 μm every other day after being exposed to 7 days of cyclic stretch. This led to an MCF as high as 4.4 mN/mm^2^, while the control group, which only received cyclic stretch, showed no significant improvement in MCF (Kensah et al., 2013). Recently, another team investigated the reaction of hiPSC-CM-based heart tissue to progressive stretch at different increments, observing that stretching at the highest rate (0.32 mm per day) generated an impressive MCF of 11.28 mN/mm^2^, a 5.1-fold increase compared to tissue stretched under fixed-length, nearly reaching the MCF of adult human hearts (15.1 mN/mm^2^). The sarcomere length also reached the degree of adult human ventricular myocytes (2.2 μm), accompanied by other evidently mature phenotypes (Lu et al., 2021). Particularly, compared to the reversible elastic deformation induced by cyclic stretch, the progressive stretch is found to actually improve longitudinal cellular growth, making it one of the most promising approaches for not only CM maturation but also the generation of large-scale hSC-CM-based heart tissues (Lu et al., 2021). The main stretch strategies as well as corresponding outcomes are summarized in Table 2.

**TABLE 2:** Stretch strategies to promote SC-CM maturation.

Cell source	Major Non-CM cell	CM proportion	Stretch strategy	Duration	MCF (mN/mm^2^)	References
hESC	∼25% HUVEC	∼50%	Static stretch	3 weeks	0.08	Tulloch et al. (2011)
	∼25% marrow stromal cells or MEF					
hESC	Unspecified	40%–50%	12%, 1.25 Hz uniaxial cyclic stretch	3 days	-	Mihic et al. (2014)
hESC and hiPSC	∼12% SMC	45%–64%	5%, 1 Hz uniaxial cyclic stretch	2 weeks	∼1.0	Ruan et al. (2015)
	∼9% EC					
hESC	Unspecified	18%	5%, 0.33 Hz uniaxial cyclic stretch	12 days	-	Shen et al. (2017)
hESC	3% FB or MSC	60%–90%	10%, 1 Hz uniaxial cyclic stretch	3 days	2.96	Zhang et al. (2017)
hESC	∼10% SMC	>90%	Progressive stretch, increment = 0.025 mm	—	2.3	Turnbull et al. (2014)
	∼5% EC					
hESC and hiPSC	∼10% FB	∼90%	Progressive stretch, increment = 0.2 mm every second day	14 days	4.4	Kensah et al. (2013)
hiPSC	Unspecified	69%	Progressive stretch, increment = 0.32 mm/d	21 days	11.28	Lu et al. (2021)

The percentage in the “Stretch Strategy” column refers to the percentage of deformation on the basis of the original cell length. CM, cardiomyocyte; EC, endothelial cells; FB, fibroblast; hESC, human embryonic stem cell; hiPSC, human induced pluripotent stem cell; HUVEC, human umbilical vein endothelial cell; MCF, maximum contractile force; MEF, mouse embryonic fibroblasts; MSC, mesenchymal stem cell; SMC, smooth muscle cell.

The mechanisms explaining the observed effects of mechanical load stimulation on hSC-CM maturation and functional integration have been partially elucidated. Studies have revealed that the upregulation of key ECM adhesion proteins, such as integrin β1 and vinculin, occurred in hESC-CMs following exposure to cyclic stretch (Zhang et al., 2017). Further investigations discovered that proteins involved in nuclear mechanotransduction, which translates mechanical signals into molecular and cellular responses, were also upregulated (YAP1 by 1.9 fold, Lamin A/C and plectin by 1.3 fold, desmin by 2.4 fold), along with an increase in nuclear size and a 1.9-fold increase in YAP nucleus localization (Song et al., 2022). These findings suggest that cyclic stretch is sensed and transmitted through integrins and focal adhesions, transduced *via* the LINC (linkers of the nucleoskeleton to the cytoskeleton) system, resulting in nuclear deformation and increased nuclear rigidity, which induces the nuclear localization of YAP and activates the Hippo/Yap pathway and downstream mechanical-related genes and cardiac marker proteins (Song et al., 2022). The aforementioned pathways are thought to regulate various properties of CMs, such as contraction properties and expression of maturation-related genes. However, further research is needed to provide more direct evidence supporting these conclusions and provide a deeper understanding of the complex mechanotransduction network.

#### 2.4.2 Fluidic load stimulation

As previously noted, the myocardium in a natural developmental process is exposed to the shear stress generated by blood flow. Therefore, it is thought that uniaxial stress alone is insufficient in representing the *in vivo* mechanical environment in which the heart develops and matures (Shen et al., 2017). In light of this, several teams have aimed at developing platforms that can replicate the natural hemodynamic environment (Shen et al., 2017; Kolanowski et al., 2020; Veldhuizen et al., 2020; Huebsch et al., 2022). To date, several reports of microfluidic systems that provide such hemodynamic fluidic stress have been proven to mature the structural and functional properties of hSC-CMs. These systems typically feature gravity- or pump-driven dynamic pulsatile flow with adjustable velocity, which creates not only shear stress but also dynamic pressure [respectively 0.71 dyn cm^−2^ and 15 mmHg, in accordance with (Kolanowski et al., 2020)]. Additionally, these systems can be designed to incorporate co-culture with niche cells (Veldhuizen et al., 2020), control of oxygen levels (Kolanowski et al., 2020), metabolic (Huebsch et al., 2022) and geometric engineering (Veldhuizen et al., 2020), thus providing multifaceted potentials to promote hSC-CM maturation. While current cell chambers in microfluidic systems are limited in size, the microfluidic systems have the potential of high-throughput culturing of CMs, unlike uniaxial stretching systems in which CMs are typically treated in the form of single cells or CM-based stripes to ensure optimal force direction (Kolanowski et al., 2020).

## 3 Electrical stimulation

Electrical stimulation is one of the most effective strategies to accelerate the maturation of SC-CMs cultivated *in vitro*, resulting in robust and sustained mature phenotypes of the contractile apparatus, Ca^2+^ handling, and electrophysiological properties, as well as other structural and functional characteristics of mature CMs. Over the years, several groups have attested that the application of electrical stimulation results in rapid maturation of SC-CMs within a relatively short period of time, thereby offering a viable strategy for the development of functional CMs, even CM-based tissue constructs (Nunes et al., 2013; Hirt et al., 2014; Eng et al., 2016; Sun and Nunes, 2017; Crestani et al., 2020; Zhao et al., 2020; Shen et al., 2022).

The electrical stimulation is applied at various stages with ranging frequencies and durations in different studies. Although it is mostly applied after differentiation has been completed, Crestani et al. reported that applying electrical stimulation during the early stages of SC-CM differentiation resulted in a more specific and mature CM phenotype (Crestani et al., 2020). Hirt and colleagues found that chronic electrical stimulation for 4 weeks was more effective in promoting SC-CM maturation than short-term stimulation (Hirt et al., 2014). The frequency of electrical stimulation is also an important factor. Specifically, different frequencies influence the adaptation of the beating rate of SC-CMs through the regulation of the *KCNH2* gene, which encodes the potassium ion channel K_v_11.1, an essential component for adaptation and contraction synchronization (Eng et al., 2016). While electrical stimulation of various frequencies is able to induce SC-CM maturation, studies have shown that for frequencies under 6 Hz, higher frequencies were associated with greater levels of structural and functional maturation (Nunes et al., 2013; Hirt et al., 2014; Sun and Nunes, 2017; Zhao et al., 2020). However, few studies have investigated frequencies above 6 Hz, as this frequency exceeds far beyond the physiological range. Additionally, researchers have investigated the effects of gradual increments in the frequency of stimulation. Notably, increasing frequencies from 1 Hz to 6 Hz instead of 3 Hz was found to induce optimal maturation (Nunes et al., 2013). In contrast, another study showed ramping up the frequency by 1 Hz weekly from 2 Hz to 6 Hz resulted in better maturation than ramping up by 0.2 Hz daily, indicating a slow step-up in stimulation frequency is more effective in maturing the SC-CMs (Zhao et al., 2020).

The utilization of electrical stimulation in conjunction with other maturation strategies can also result in favorable hSC-CM maturation. When cultured in high Ca^2+^ concentration, SC-CMs that simultaneously undergo electrical stimulation exhibit better contractile force and calcium handling properties, indicating improved maturation (Shen et al., 2022). Researchers have developed an engineered system, referred to as “Biowire,” which cultured cells in a collagen type I gel along a rigid template suture and applied electrical stimulation at increasing frequencies. The Biowire system not only provided electrical stimulation but also created a geometric environment conducive to the growth of immature SC-CMs into 3D structures. Biowire has been shown to improve the structural maturation of SC-CMs, induce physiologic hypertrophy, enhance sarcomere maturation, and promote electrophysiological properties. However, it is worth noting that the suture size of Biowire is relatively small due to the limited distance of oxygen supply (Nunes et al., 2013; Sun and Nunes, 2017). Additionally, a polystyrene-based heart-on-a-chip platform incorporating electrical stimulation, non-myocyte cells, and other micro-environmental cues, has demonstrated effective promotion of hSC-CM maturation (Zhao et al., 2020).

Taken together, electrical stimulation is a relatively accessible and effective strategy for *in vitro* hSC-maturation. However, researchers have also discovered a link between chronic electrical pacing and inflammatory response, possibly as a mechanism of self-protection against pacing-induced oxidative stress (Hirt et al., 2014). In conclusion, electrical stimulation holds significant potential for generating functionally and structurally mature CMs for disease modeling, drug screening, and even possible clinical applications. Future studies may pinpoint more specific mechanisms by which hSC-CMs respond to electrical stimulation. The optimal magnitude, frequency, the initiation and duration time also demand further study. Lastly, significant disparities still exist between adult cardiac tissue and cultured hSC-CM, even after electrical stimulation. This gap may be bridged by systems that provide a combination of various maturation-promoting strategies, such as Biowire.

## 4 Co-culture and cell-cell interaction

Pinto et al. have previously reported a comprehensive landscape of the composition of the heart. An adult mouse heart contains only 30% contractile CMs, while the rest of the non-CM parts are comprised of ∼60% endothelial cells (ECs), ∼30% cardiac fibroblasts (CFs), and ∼10% leukocytes (Pinto et al., 2016). With the proliferation of non-CMs, the proportion of CMs gradually decreases after birth. This reveals the potential effects of non-CMs on CM maturation through intercellular crosstalk.

The traditional SC-CM differentiation protocols aimed at obtaining a pure population of SC-CMs. In more recent studies, inspired by the population characteristics of the adult mouse heart, non-CMs such as ECs, CFs, and mesenchymal stem cells (MSCs) have been introduced to the environment. The co-culturing method further increased the complexity and created the opportunity for cell-cell crosstalk, and has been shown to promote SC-CM maturation (Kim et al., 2010). The mechanisms underlying the assistance from non-CMs may include non-CM-derived paracrine factors and direct cell-cell interactions. For example, MSCs induce SC-CMs maturation *via* paracrine of vascular endothelial growth factor (VEGF), basic fibroblast growth factor (bFGF), stromal cell-derived factor 1 (SDF-1), and granulocyte-macrophage colony-stimulating factor (GM-CSF) (Yoshida et al., 2018). Additionally, CFs and ECs are capable of secreting essential components of cardiac ECM, such as collagen and fibronectin, to provide more favorable stiffness for SC-CM maturation (Yu et al., 2018; Dunn et al., 2019). Notably, coculturing SC-CMs with non-CMs, such as CFs, offers a promising solution for one of the central barriers for translational application, graft-related arrythmia. This side effect is attributed to the unsynchronized contraction, which may result from inadequate cell-cell interaction among SC-CMs. A study has shown that coculturing SC-CMs with CFs can enhance the formation of connexin 43 gap junctions, thereby promoting intercellular communication (Giacomelli et al., 2020).

## 5 Biochemical and molecular biological cues

### 5.1 Hormones

The idea of introducing hormones into *in vitro* maturation of SC-CMs is based on the knowledge of two events that take place simultaneously: the rising levels of tri-iodothyronine (T3) and glucocorticoids, and the initial emergence of phenotypes that indicate cardiac maturation (Rog-Zielinska et al., 2013; Li et al., 2014; Rog-Zielinska et al., 2015). A subsequent study confirmed that treating SC-CMs with 20 ng/mL of T3 for 1 week enhanced the cell size, sarcomere length, calcium handling, and contractile kinetics (Yang et al., 2014). A combined supplement of 100 nmol/ml T3 and 1 μmol/mL dexamethasone (Dex) for about 15 days further promoted t-tubule formation (Parikh et al., 2017). Using a similar strategy, two recent studies have also identified the maturation of contractile and electrophysiological properties under T3 and Dex treatment (Huang et al., 2020; Wang et al., 2021). Apart from T3 and Dex, Sim et al. (2021) have recently uncovered the important role of progesterone receptors on CM maturation using multi-omics methods. An extra supplement of 10 μM progesterone for 48 h can augment cardiac contractility and reduce the spontaneous contraction rate in both SC-CMs and human cardiac organoids, indicating a new pathway for *in vitro* maturation of SC-CMs.

### 5.2 Transcription factors

Identifying the link between diverse phenotypic changes and downstream transcription factors provides more possible ways for *in vitro* regulation. In the context of CM maturation, most of them are associated with metabolic processes.

Nuclear receptors (NRs), which are important in sensing fat-soluble molecules (hormones, vitamins, fatty acids (FAs), *etc.*), have crucial roles in CM maturation. For example, T3 and Dex are ligands of thyroid hormone receptors (encoded by *THRA* and *THRB*) and glucocorticoids receptor (encoded by *NR3C1*) respectively. Receptors of sex steroids have also been investigated for CM maturation. Adding extra testosterone or progesterone, ligands of the androgen receptor (encoded by *AR*) and progesterone receptor (encoded by *PGR*), has demonstrated positive effects on SC-CM maturation (Sim et al., 2021). In addition, estrogen-related receptors (ERRs), a group of orphan NRs, can downstream activate crucial genes involved in fatty acid oxidation (FAO), tricarboxylic acid cycle, and oxidative phosphorylation, promoting the switch to aerobic metabolism (Dufour et al., 2007). Another study further defined the critical role of ERRα and ERRγ in SC-CM maturation (Sakamoto et al., 2020). However, since the ligands targeting ERRs are still uncharacterized, it is difficult to manipulate ERRs signaling to promote SC-CM maturation.

Peroxisome proliferator-activated receptor (PPAR) is another family of transcription factors that are activated by a series of ligands, regulating various cell activities. The effects of modulating PPARs will be discussed later in the section of metabolic engineering. Here, we dive further into the PPAR coactivator 1 α/β (PGC1α/β, encoded by *PPARGC1* and *PPARGC2*), which can directly interact with PPARs and ERRs, acting as a master regulator of mitochondrial biogenesis and oxidative respiration (Dorn et al., 2015). Since PGC1α is a downstream targeting gene of the AMPK (adenosine 5′-monophosphate (AMP)-activated protein kinase) signaling pathway, AMPK activation results in upregulated PGC1α expression, improvement in mitochondrial function, and increase in mitochondrial fusion in SC-CMs (Ye et al., 2021). Dual treatment with two PPAR/PGC1α activators, asiatic acid and GW501516 (a PPARβ/δ agonist) successfully induced the maturation of SC-CMs as well (Chirico et al., 2022). Another PGC1α activator ZLN005 showed more effectiveness in increasing sarcomere length and improving calcium handling in SC-CMs (Liu et al., 2020). Notably, treatment with some alkaloids, like tomatidine also functions through PGC1α to enhance SC-CM maturation (Kim et al., 2022).

Nuclear factor erythroid 2 p45-related factors 2 (NRF2, encoded by *NFE2L2*) is known as a key regulator of cellular redox homeostasis (Dinkova-Kostova and Abramov, 2015). In SC-CMs, Ramachandra’s team first discovered a high expression level of NRF2 after fatty acid (FA) treatment (Ramachandra et al., 2018). A recent study also demonstrated the indispensable role of NRF2 in SC-CM maturation using genetic manipulation (Zhang et al., 2021). Significant decrease in cell perimeter, cell area and sarcomere length were observed after knocking down NRF2 by targeting siRNA. Also, it is known that the activity of NRF2 is regulated by an E3 ubiquitin ligase complex composed of cullin-3 (Cul3) and Kelch-like ECH-related protein 1 (KEAP1). Thus, inhibiting Cul3 and KEAP1 with small molecules may activate NRF2, then promoting the maturation of SC-CMs.

### 5.3 Signaling pathways

Induction of SC-CM maturation by manipulating particular signaling pathways is a relatively easy approach since the activation or inhibition of certain kinases has been a widely established laboratory method. Despite the clear characterization of AMPK signaling in the metabolic maturation of CMs, an up-to-date study discovered the MAPK/PI3K/AKT pathway is also involved in this process. An extra supplement of 10 μM PD0325901 (MEK1/2 inhibitor) and 5 μM SB203580 (p38/PDK1 inhibitor) has been shown to be an efficient way to improve SC-CM maturation in morphology and expression of adult isoform sarcomeric proteins, which inspired further research to focus on screening relevant pathways that can induce SC-CM maturation (Garay et al., 2022).

### 5.4 RNAs

MicroRNAs (miRNAs) have been defined as regulators of cardiac development. Several routes to induce SC-CM maturation by modulating particular miRNAs have emerged. Delivering a miRNA cocktail (miR-125b-5p, miR-199a-5p, miR-221, and miR-222) to SC-CMs brings about improvement in several aspects, including sarcomere alignment, Ca^2+^ handling, resting membrane potential, and the expression of maturation markers (Lee et al., 2015). One pioneering research also found the let-7 family of miRNA is closely related to CM maturation. After overexpression of let-7 family members in SC-CMs, cell size, sarcomere length, force of contraction and respiratory capacity were improved significantly (Kuppusamy et al., 2015). Recently, a bioinformatic analysis of the SC-CM dataset uncovered the positive effect of miR-124 on SC-CM maturation (Muñoz et al., 2022).

The strategies mentioned above that promote hSC-CM maturation through the manipulation of biochemical and molecular biological cues offer direct, precise, and immediate control over the fundamental processes that govern cellular activity, particularly the maturation of SC-CMs, at a relatively low cost. The development and application of biochemical or molecular regulators can also shed light on the intricate intracellular network that regulates CM maturation and various phenotypes. This may inspire the exploration of new or improved maturation strategies and aid in the development of disease models and potential treatment plans, though the effective and selective application of molecules to target SC-CMs *in vivo* may require further investigation.

## 6 Metabolic engineering

Native cardiomyocytes exhibit a unique metabolic and bioenergetic shift in their natural process of development from fetal CMs to adult CMs to meet their high demands for energy metabolism (Gaspar et al., 2014). Specifically, the primary source of adenosine triphosphate (ATP) undergoes a distinct transition from anaerobic glycolysis, which primarily utilizes glucose and product lactic acid in fetal CMs, to fatty acid oxidation (FAO) in adult CMs, for FA has the highest energy density among all common metabolic substrates (Gaspar et al., 2014). The gradual glycolysis-to-FAO shift and increased oxidative capacity have also been observed in hiPSC-CMs exposed to prolonged culture *in vitro* (Emanuelli et al., 2022) The developmental changes of energy metabolism in CMs are consistent with the observed hypoxia-induced expression of hypoxia-inducible factor 1α (HIF-1α) in fetal CMs, which leads to increased expression of genes associated with glycolysis. Conversely, adult CMs exhibit a gradual downregulation of HIF-1α and an increase in PPAR expression, a family of key factors that regulate FAO, in addition to other proteins involved in FA transportation (Gentillon et al., 2019; Feyen et al., 2020; Wickramasinghe et al., 2022). Moreover, differences in the distribution and number of mitochondria also occur, with immature CMs possessing peripherally or nuclei-adjacent scattered mitochondria, while adult CMs exhibit a more evenly dispersed distribution throughout the cell (Gherghiceanu et al., 2011) Given the distinct metabolic features observed in adult CMs, it has been hypothesized and subsequently demonstrated that metabolic engineering which simulates the metabolic patterns and/or environment of developing or mature CMs promotes the maturation of hSC-CMs.

### 6.1 Metabolic substrate engineering

The most direct and well-explored metabolic engineering approach to maturing newly differentiated SC-CMs is to create a metabolic environment recapitulating the native environment in which CMs develop and mature. Previous studies have reported a serum FA concentration of 300 μM in human newborns (Makinde et al., 1998). However, the commonly used media for CM differentiation, namely, RPMI-B27-insulin media, contains less than 10 μM of FA (Yang et al., 2019). The glucose-rich microenvironment provided by media like RPMI-B27-insulin can also hinder the metabolic maturation of CMs by inhibiting FAO (Nakano et al., 2017; Feyen et al., 2020). Based on these findings, various low-glucose, FA-supplemented maturation media have been developed to improve the structural, functional, pharmacological, and especially metabolic maturation of hESC- and hiPSC-CMs (Correia et al., 2017; Lin et al., 2017; Horikoshi et al., 2019; Yang et al., 2019; Feyen et al., 2020; Knight et al., 2021; Huebsch et al., 2022).

These basal media are typically supplemented with various FAs, along with other maturation-promoting molecules mentioned in Section 5. Table 3 summarizes the media used in the aforementioned studies, as well as the maturation effect assessed by parameters such as the circularity index, PPAR expression, maximum contractile force (MCF), and action potential duration (APD). In recent studies, researchers have further developed FA-albumin complexes to avoid lipid toxicity (Yang et al., 2019) and incorporated micropatterning method to add geometric control (Knight et al., 2021), resulting in improved maturity. Interestingly, glucose starvation alone, apart from FA supplement, has also been shown to promote CM maturation on a single-cell scale (Ni et al., 2021).

**TABLE 3:** The medium used for SC-CM maturation.

Major supplement	CM purity	Circularity index	PPAR expression	MCF (mN/mm^2^)	APD (ms)	References
10 mM galactose, 100 µM OA, 50 μM PA	>80%	∼0.3	increased	0.4	APD_50_: 580	Correia et al. (2017)
fatty acid + T3	>90%	—	increased by 5-fold	—	APD_50_: 450	Lin et al. (2017)
LA-OA-albumin	98%	∼0.5	increased by 2-fold	—	—	Horikoshi et al. (2019)
105 μM PA-albumin, 81 μM OA-albumin, 45 μM LA-albumin	>80%	∼0.5	—	14 (nN/cell)	APD_50_: 282	Yang et al. (2019)
L-lactate, lipid-rich BSA	95%	—	—	—	increased	Feyen et al. (2020)
galactose, OA, LA	>75%	∼0.2	increased	33	—	Knight et al. (2021)
10 mM galactose, 125 µM OA, 100 μM PA	80%	—	—	∼5	APD_80_: 420	Huebsch et al. (2022)

APD_x_, refers to action potential duration at x% repolarization. BSA, bovine serum albumin; CM, cardiomyocyte; LA, linoleic acid; MCF, maximum contractile force; OA, oleic acid; PA, palmitic acid; PPAR, peroxisome proliferator-activated receptor; T3, tri-iodothyronine.

### 6.2 Metabolic pathway engineering

With a deeper investigation of FA-supplement-related hSC-CM maturation, PPAR and AMPK have been identified as key pathways highly responsive to FA treatment, and responsible for FA oxidation (Horikoshi et al., 2019; Yang et al., 2019; Murphy et al., 2021). So far, several teams have reported promising maturation outcomes by directly activating PPAR and AMPK signal pathways.

PPAR is a family of transcription factors that are activated by various ligands, including FAs and hormones. There are three isoforms of PPAR (α, β/δ, and γ), with PPARα being the most abundantly expressed and active isoform during hSC-CM differentiation (Wickramasinghe et al., 2022). Studies have shown that activation of PPARα enhances FAO, the expression of genes involved in FA transportation and oxidation, and other hallmarks of CM maturation (Gentillon et al., 2019; Funakoshi et al., 2021; Lopez et al., 2021; Murphy et al., 2021). However, most studies either applied PPARα agonists at a high concentration or in conjunction with other modulation factors such as T3 and Dex (Gentillon et al., 2019; Funakoshi et al., 2021). PPARα agonist treatment alone has been reported to be insufficient in inducing hSC-CM maturation, possibly due to its inherent abundance and high activity (Wickramasinghe et al., 2022). In a recent study (Wickramasinghe et al., 2022), researchers discovered that the maturation and metabolic effects of PPAR activation can be isoform-specific. For instance, the increase of myofibril alignment and cell size were only observed when PPARδ, instead of PPARα or PPARγ, was activated. More intriguingly, compared with PPARα, the activation of PPARδ resulted in an even more intensive upregulation of the FAO gene network and glycolysis-to-FAO shift, yielding improved electrical and contractile properties.

AMPK, on the other hand, is a protein kinase that is highly sensitive to cellular energy status that regulates various cellular metabolic processes, including FAO. The activation of AMPK has also been shown to improve FAO and CM maturation (Ye et al., 2021), which, in conjunction with the finding that expression of CD36 is upregulated after PPARα activation, may provide new insights into the complex network coupling metabolic shifts with CM maturation. Finally, inhibition of the HIF-1α pathway, a process occurring in the natural development of native CMs, also improved FAO and CM maturation (Hu et al., 2018; Gentillon et al., 2019).

By directly manipulating the key metabolic proteins and pathways essential for hSC-CM maturation, it becomes possible for researchers to engineer the metabolic status of CMs more directly, precisely, and effectively. This not only promotes hSC-CM maturation but also allows for further investigation into molecular pathways and mechanisms that couple metabolic status with other maturation hallmarks in hSC-CMs. Metabolic pathway engineering may also offer more options for designing disease models for metabolic syndromes that affect myocardium, even providing insights into precise treatment. However, this approach is obviously more challenging as it requires a deeper understanding of metabolic features and signaling pathways.

In summary, compared with other strategies for hSC-CM maturation, metabolic programming is particularly valuable for its ease of accessibility and cost-effectiveness. Furthermore, it has the potential to generate large-scale disease models and biocompatible tissues. Additionally, in light of the unique metabolic shift and property of cardiomyocytes, the metabolic cues themselves can serve as a method for purifying hSC-CMs, resulting in not only improved maturity but also increased purity. The fact that metabolic cues are compatible with a wide range of other maturation methods also expands the potential scope for their application. However, it is important to note that metabolic programming alone is not yet capable of producing fully mature hSC-CMs (Yang et al., 2019). Therefore, it is crucial to explore multiple complex maturation approaches that involve metabolic control in the future. Another unaddressed issue is the complex mechanism network linking the metabolic engineering and the maturation phenotype. While a few research teams have proposed that PPAR activation may contribute to hSC-CM maturation *via* YAP1 and SF3B2 (Murphy et al., 2021), or *via* CD36, which not only transports FAs but also regulates Ca^2+^ signal transduction and AMPK activation (Gentillon et al., 2019), the specific and comprehensive mechanisms remain an area in need of further investigation.

## 7 Prolonged culture

Prolonged culture is a direct method for enhancing the maturation of hSC-CMs. In practice, this approach is often utilized in conjunction with other methods aimed at engineering the maturation of CM. To assess the impact of long-term culture on maturity, researchers must conduct prolonged follow-up studies to detect changes that occur over time. So far, researchers have observed various markers indicating CM maturation that appear as a result of prolonged culture, such as increased cell size, anisotropy, density, and alignment of myofibrils (Lundy et al., 2013; Dai et al., 2017). Notably, CMs cultured for 1 year exhibited evident M-band, indicating the maturation of the sarcomere, though the expression of genes related to M-band was still inferior compared to adult human hearts (Kamakura et al., 2013; Lundy et al., 2013). In terms of contractility, contractile kinetics of late-stage hSC-CMs were lower, and the beating rate decreased, but it has shown no difference in spontaneous contraction rate (Kamakura et al., 2013; Lundy et al., 2013). The electrophysiology, Ca^2+^ transient, cardiac gene expressions, as well as the functionality and distribution of mitochondria, have all been reported to mature in hSC-CMs exposed to prolonged culture, yet none reached the levels of adult human hearts (Kamakura et al., 2013; Lundy et al., 2013; Dai et al., 2017). A recent study combining prolonged culture with a system that provided mechanical stimulation also reported more mature hSC-CM (Shen et al., 2017).

The aforementioned studies have provided compelling evidence that hSC-CMs can mature *in vitro* as they are cultured for an extended period of time, despite the drastic differences between the *in vitro* and *in vivo* environments in which CMs are developed. However, hSC-CMs that undergo prolonged culture alone exhibit limited maturation compared to developing or adult human hearts, possibly due to the absence of certain fundamental microenvironmental cues that remain to be revealed in future studies. Practically, it is also necessary to consider the cost-effectiveness as well as the risk of contamination during prolonged culture. Given the current studies and evidence, prolonged culture is more likely to be utilized as an incidental strategy in conjunction with other methods aiming at promoting hSC-CM maturation.

## 8 3D cardiac tissue constructs

3D cardiac tissue constructs are functional platforms for housing, culturing, and maturing cardiomyocytes *in vitro*. Such constructs include cardiac organoids (COs) that rely on the intrinsic capacity of human organs for self-assembly, engineered heart tissues (EHTs) or 3D microtissues that are artificially oriented for assembly, and a variety of small cardiac patch platforms with scaffolds (Shadrin et al., 2017; Schaefer et al., 2018; Varzideh et al., 2019; Giacomelli et al., 2020; Veldhuizen et al., 2020; Kawai et al., 2022; Ramirez-Calderon et al., 2022). In these systems, hSC-CMs undergo hypertrophy and gradually exhibit some maturation indicators in terms of ultrastructure, electromechanical properties, and biochemical metabolism. Additionally, these systems can drive the continuous contraction and spontaneous beating of CMs throughout the system (Eng et al., 2016; Ulmer et al., 2018; Goldfracht et al., 2019; Varzideh et al., 2019; Giacomelli et al., 2020; Veldhuizen et al., 2020).

In recent years, the use of biomimetic collagen hydrogels derived from decellularized animal tissues as dynamic or fluidic ECMs for the construction of 3D cardiac tissue constructs has gained traction as a means of embedding and implanting CMs and various supporting cells derived from hESCs or hiPSCs (Hochman-Mendez et al., 2020; Veldhuizen et al., 2020). This is owing to the fact that these optimized 3D environments exhibit a superior promoting effect on the maturation of the morphological structure and physiological function of hSC-CMs compared to conventional 2D culture (Thavandiran et al., 2013; Goldfracht et al., 2019; Karbassi et al., 2020). Many studies have found that the topology and mechanical properties of cultures in 3D environments are more akin to the corresponding native heart tissue, and within as little as 2 weeks of culture, 3D culture is able to promote the sarcomere length of hSC-CMs to a degree comparable to adult CMs. Furthermore, various indicators of maturity have also been observed in hSC-CMs in 3D systems *in vitro* (Zhang et al., 2013; Ahmed et al., 2020). While these *in vitro* 3D cardiac tissue constructs may not fully reproduce the entire complex structure of the heart *in vivo*, they are still able to mimic the structure and function of native heart tissue to a certain extent under the synergistic effect of electrical stimulation, mechanical stimulation, and biochemical stimulation (Hirt et al., 2014; Ahmed et al., 2020; Karbassi et al., 2020).

In successful 3D culture models, the unique niche in which hSC-CMs reside is often constructed concomitantly with a complex ECM (Thavandiran et al., 2013). Under the native cardiac environment, the interplay and crosstalk between CMs, supporting cells, ECM, and microvessels constitute a large and complex network, which is crucial for maintaining the homeostasis of the tissue microenvironment and promoting the survival and maturation of CMs (Varzideh et al., 2019; Giacomelli et al., 2020; Veldhuizen et al., 2020). Studies have substantiated that supporting cells such as cardiac fibroblasts and cardiac endothelial cells form a tri-cellular combination with CMs, and this tri-cellular interaction and crosstalk may mediate the intracellular cyclic AMP pathway through direct cell-cell contact or gap junctions, thereby facilitating CM maturation and maintaining the rhythmicity and synchronous contraction of CMs (Giacomelli et al., 2020; Kahn-Krell et al., 2022). Furthermore, mimicking *in vitro* myocardial infarction environment and stress status seems to be able to induce vascularization in microtissues, thus allowing the CM niche to acquire sufficient nutrients and multiple growth factors in EHTs at physiological thickness, and although vascular structures resembling mature cardiac tissue may not be a prerequisite for the development of EHTs, this is found to create favorable conditions for the development and maturation of hSC-CMs (Shadrin et al., 2017; Schaefer et al., 2018; Varzideh et al., 2019; Giacomelli et al., 2020; Mousavi et al., 2022). The investigation of specific mechanisms underlying cell-cell, cell-ECM interactions in the cardiac microenvironment remains a major focus of future evidence-based medicine directions (Thavandiran et al., 2013).

In light of the fact that native human CMs are difficult to self-repair after injury, the study of 3D cardiac tissue constructs holds great promise for the creation of improved *in vitro* mini or large substitutes for the human heart, with the potential to facilitate investigations into the pathogenesis of cardiovascular diseases and the optimal criteria of organ modeling (Eng et al., 2016; Goldfracht et al., 2019; Varzideh et al., 2019; Giacomelli et al., 2020; Veldhuizen et al., 2020). Notably, if the safety and efficacy of EHTs and COs can be established through rigorous testing in large animal models, these 3D cardiac tissue constructs may offer new therapeutic possibilities for the treatment of myocardial infarction and heart failure (Schaefer et al., 2018; Kawai et al., 2022). For example, the utilization of cardiac patch implantation has demonstrated encouraging results in a 68-year-old patient with advanced heart failure, despite reports of observed cardiac arrhythmias following orthotropic heart transplantation (Shadrin et al., 2017; Schaefer et al., 2018). In addition, 3D hiPSC-CM organoids have also shown great promise as platforms for disease modeling and drug screening (Sharma et al., 2013). Future studies will evolve in designing 3D cardiac tissue constructs capable of electromechanical integration with host CMs and deep inosculation with host vasculature. Alongside is the ongoing exploration of 3D constructs, such as ones with increased thickness of the myocardial wall, to generate novel protocols which enhance the maturity of stem cell-derived cardiomyocytes *in vitro* (Eng et al., 2016; Guo and Pu, 2020; Sun et al., 2020; Mousavi et al., 2022).

## 9 *In vivo* implantation

*In vivo* implantation has been shown to enhance the maturation of hSC-CMs, evolving alongside the rapidly advancing 3D culture and 3D printing technologies (Ahmed et al., 2020). When implanted into rat heart tissue, hSC-CM grafts exhibit a relatively mature phenotype and adult-like morphology within 2–3 months, compared to the full year required for *in vitro* culture (Kadota et al., 2017; Ahmed et al., 2020; Guo and Pu, 2020). A recent study employing 3D printing technology has successfully engrafted EHT in mice with congenital immunodeficiency and observed more mature hSC-CMs (Kawai et al., 2022). Furthermore, a study in which COs were heterotopically implanted into the peritoneal cavity of mice also demonstrated multiple indicators of CM maturation (Varzideh et al., 2019). Notably, whether in the form of EHT or CO, hSC-CMs within these cardiac organoid systems, which incorporate both endothelial and fibroblast components, exhibit a more mature phenotype compared to those in conventional 2D-CM culture. Several studies utilizing *in vivo* implantation have also observed vascularization in small grafts like cardiopatches or cardiac organoids, ensuring smooth engraftment and nutrient supply of hSC-CMs in the setting of infarct ischemia or heart failure (Shadrin et al., 2017; Sun et al., 2020). These aforementioned changes of *in vivo* implanted hSC-CMs may be attributed to the interaction with niche cells, such as mesenchymal cells, through direct contact or paracrine secretion (Zhang et al., 2017; Varzideh et al., 2019).

While successful outcomes have been reported, a critical view must be taken to evaluate the potentials of *in vivo* implantation for hSC-CM maturation, as this methodology has unique advantages and weaknesses. To begin with, the whole maturation process of CMs may involve yet unknown cellular communication and a complex intra-cardiac microenvironment which cannot be fully replicated *in vitro*, highlighting the unique and irreplaceable advantages of *in vivo* implantation (Ahmed et al., 2020). In addition, *in vivo* implantation allows researchers to advance a deeper understanding of host-graft interactions, how such interactions induce hSC-CM maturation, and how hSC-CM maturation status affects the restoration of normal function in host hearts within animal models such as myocardial infarction. With these advantages being said, *in vivo* implantation yet still faces unique challenges that need to be addressed. To the very least, the involvement of an undefined, xenogenic, dynamic environment raises safety concerns when potential clinical applications are taken into account. Moreover, multiple effective *in vitro*, animal-free methods have produced structurally and functionally matured hSC-CMs, while current *in vivo* methodology has reported no significant outcome improvement in comparison with *in vitro* strategies. Ergo, *in vivo* implantation solely for harvesting matured hSC-CMs would be deemed unnecessary and ethically questionable, particularly when referring to the principles of the 3Rs (Reduction, Replacement, and Refinement) in animal studies.

Therefore, the future direction in the practice of *in vivo* implantation of SC-CMs should be clinical application-guided, and conducted in accordance to the principles of the 3Rs. This may entail investigating the complex mechanism of host-graft interaction that regulate the graft maturation, therapeutic and side effects, immune responses, and the interplay between these events. Large-scale COs may also be designed based on preclinical studies in large animals, which necessitates the development of exploratory studies which focus on establishing transplantation protocols of hSC-CMs that apply to non-human primates, as studies suggest that transplanted hSC-CMs exhibit more pronounced maturation in the hearts of non-human primates compared with those transplanted in rat hearts (Laake et al., 2007; Shadrin et al., 2017; Karbassi et al., 2020). Meanwhile, it is also crucial to explore and adopt suitable immune tolerance strategies to mitigate the immune rejection brought about by hiPSC-CM transplantation, further creating an optimal cardiac environment for the growth and maturation of hSC-CMs, thus paving way for future clinical translation (Dai et al., 2007).

## 10 Discussion

In light of the rapid development of stem cell technology, human stem cell-derived cardiomyocytes have emerged as an extremely promising platform for cardiac disease modeling, drug screening, and a potential source for myocardium regeneration. Despite the promising vision, researchers worldwide have been faced with the challenge that newly differentiated SC-CMs are structurally and functionally immature, exhibiting various phenotypes that resemble neonatal CMs, rather than adult CM, greatly impeding the next-step application (Liu et al., 2018; Reilly et al., 2022). To address this limitation, a wide range of strategies have been developed to promote and engineer SC-CM maturation, each with distinct advantages and limitations.

Essentially, the maturation strategies are engineered to recapitulate the mechanical, electrical, biochemical, and/or cellular environment during *in vivo* post-born heart development. For instance, several research teams have successfully induced structural and functional maturation of SC-CMs by engineering the geometry of substrate according to the morphology and/or alignment of mature cardiomyocytes (Rao et al., 2013; Salick et al., 2014; Khan et al., 2015; Ribeiro et al., 2015; Rodriguez et al., 2019; Yu et al., 2019; Strimaityte et al., 2022), or providing substrate stiffness to mimic the physiological stiffness of adult myocardium (Feaster et al., 2015; Ribeiro et al., 2015; Herron et al., 2018; Wheelwright et al., 2018; Rodriguez et al., 2019). In addition, native CMs are developed in a highly complex cellular environment comprised of a number of non-CM cells, such as cardiac fibroblasts and epithelial cells, and the ECM secreted by resident cells with mixed chemical components and microarchitectures. Thereby, researchers have also explored the effects of decellularized ECM harvested from cadaver hearts (Rienks et al., 2014; Fong et al., 2016; Sachs et al., 2017; Goldfracht et al., 2019; Yu et al., 2019; Hochman-Mendez et al., 2020; Mesquita et al., 2021; Xiao et al., 2021) and co-culturing with niche cells (Kim et al., 2010; Yoshida et al., 2018; Yu et al., 2018; Dunn et al., 2019), all resulting in the maturation of SC-CMs. Electrical stimulation that acts as pacemaker cells plus the stretch and fluidic stimulations that mimic the stretch force and hemodynamic shear force produced by CM contraction and blood flow, respectively, all have been shown to enhance SC-CM maturation. The maturation of SC-CMs has also been reported by providing them with a FA-enriched, glucose-depleted environment that fit the metabolic characteristics of mature CMs (Correia et al., 2017; Lin et al., 2017; Horikoshi et al., 2019; Yang et al., 2019; Feyen et al., 2020; Knight et al., 2021; Huebsch et al., 2022), or activating PPARs that facilitate fatty-acid oxidation (Gentillon et al., 2019; Lopez et al., 2021; Wickramasinghe et al., 2022).

While such a great many maturation strategies have been proposed and subsequently confirmed, several questions remain to be answered in future studies. To begin with, most studies either failed to reveal the specific mechanisms by which engineered conditions interact with cellular and molecular activities, resulting in SC-CM maturation. To date, the mechanisms for mechanical stretch (Song et al., 2022) and signal pathway regulation have been partly illustrated, yet more direct evidence is necessary to support the authors’ conclusions. In addition, considering the extremely complex environment of *in vivo* heart development which encompasses various cues subject to dynamic changes, it is difficult, if not impossible, and probably even unnecessary to fully recreate the native developing environment *in vitro* (Ahmed et al., 2020)*.* Therefore, it is important to pinpoint which cues are imperative and most cost-effective, in order to achieve optimal maturation effects with controllable strategies. Since most approaches cannot induce fully matured SC-CMs with phenotypes identical to adult CMs, it may be helpful to engineer a complex system that provides control in various parameters and cues that regulate CM maturation. The Biowire system is a good example, incorporating geometric engineering, electrical stimulation, and niche cells to form a 3D construct (Nunes et al., 2013; Wang et al., 2020). Several other cardiac patch systems have also demonstrated great potential for combining multiple engineering strategies (Zhang et al., 2013; Hochman-Mendez et al., 2020). Last but not least, the utilization of xenogenic or chemically undefined materials such as pig heart-derived dECM, or chemicals that have not yet been proven biocompatible, can also hinder clinical applications. Therefore, alternative biocompatible, xeno-free materials may be worth investigating. Exploratory experiments on animals such as non-human primates to test the biosafety as well as the efficacy of CMs or CM-based 3D constructs may also pave the way for clinical scenarios. The distinct advantages and challenges of major strategies have been summarized in Table 4.

**TABLE 4:** Major advantages and limitations of several maturation strategies.

Strategy	Advantage	Limitation
Geometric engineering	1. High adjustability	The need for bioengineering technology and equipment
	2. High compatibility with other maturation strategies	
Substrate stiffness & chemically engineered ECM engineering	1. Low cost and easy accessibility	Unclear biocompatibility and potential degradability
	2. High compatibility with other maturation strategies	
dECM	Representation of relatively comprehensive chemical and secretory environment of native cardiac development	The use of xenogenic and chemically poor-defined materials
Stretch load	1. Rapid maturation with high efficiency	1. Limited throughput
	2. Promotion of cell growth, with the potential to generate large-scale SC-CM-based tissue constructs	2. Possibility of sarcomere break and cell damage
Fluidic load	1. Potential for high-throughput culture	1. Currently limited size of the fluidic system
	2. Unique hemodynamic stress resembling native cardiac development	2. The need for specially-designed equipment
Metabolic and biochemical/molecular biological engineering	1. Low cost and easy accessibility	Potential dosage-dependent toxic side effects
	2. High compatibility with other maturation strategies	
	3. Possible screening effect	
*In vivo* implantation	1.Comprehensive chemical, mechanical and secretory environment of native cardiac development	1. Ethical concerns to the necessity of using experimental animals
	2.Close relation to clinical application	2. Safety concerns

ECM, extracellular matrix; SC-CM, stem cell-derived cardiomyocyte.

## Data Availability

The original contributions presented in the study are included in the article/supplementary material, further inquiries can be directed to the corresponding authors.

## Glossary

2D: two-dimensional
3D: three-dimensional
AMPK: adenosine 5′-monophosphate (AMP)-activated protein kinase
APD: action potential duration
ATP: adenosine triphosphate
BCC: binary colloidal crystal
bFGF: basic fibroblast growth factor
BSA: bovine serum albumin
CF: cardiac fibroblast
CM: cardiomyocyte
CO: cardiac organoid
Cx43: connexin 43
dECM: decellularized extracellular matrix
Dex: dexamethasone
EC: endothelial cells
ECM: extracellular matrix
EHT: engineered heart tissue
ERR: estrogen-related receptor
FA: fatty acid
FAO: fatty acid oxidation
FB: fibroblast
GM-CSF: granulocyte-macrophage colony-stimulating factor
hESC: human embryonic stem cell
hESC-CM: human embryonic stem cell-derived cardiomyocyte
HIF-1α: hypoxia-inducible factor 1α
hiPSC: human induced pluripotent stem cell
hiPSC-CM: human induced pluripotent stem cell-derived cardiomyocyte
hPSC: human pluripotent stem cell
HUVEC: human umbilical vein endothelial cell
KEAP1: Kelch-like ECH-related protein 1
LA: linoleic acid
LDHA: lactate dehydrogenase A
LINC: linkers of the nucleoskeleton to the cytoskeleton
MCF: maximum contractile force
MEF: mouse embryonic fibroblast
miRNA: micro ribonucleic acid
MSC: mesenchymal stem cell
NR: nuclear receptor
NRF2: nuclear factor erythroid 2 p45-related factors 2
OA: oleic acid
PA: palmitic acid
PAA: polyacrylamide
PCL: polycaprolactone
PDMS: polydimethylsiloxane
PEG: polyethylene glycol
PGC1α/β: PPAR coactivator 1 α/β
PLGA: polylactide-co-glycolide
PPAR: peroxisome proliferator-activated receptor
RyR: ryanodine receptor
SC-CM: stem cell-derived cardiomyocyte
SDF-1: stromal cell-derived factor 1
SDS: sodium dodecyl sulfate
SC-EC: stem cell-derived endothelial cell
SERCA: sarcoplasmic/endoplasmic reticulum calcium ATPase
SMC: smooth muscle cell
SR: sarcoplasmic reticulum
T3: tri-iodothyronine
VEGF: vascular endothelial growth factor

## References

[B1] Afzal J. Liu Y. Du W. Suhail Y. Zong P. Feng J. (2022). Cardiac ultrastructure inspired matrix induces advanced metabolic and functional maturation of differentiated human cardiomyocytes. Cell Rep. 40, 111146. 10.1016/j.celrep.2022.111146 35905711

[B2] Ahmed R. E. Anzai T. Chanthra N. Uosaki H. (2020). A brief review of current maturation methods for human induced pluripotent stem cells-derived cardiomyocytes. Front. Cell Dev. Biol. 8, 178. 10.3389/fcell.2020.00178 32266260 PMC7096382

[B3] Aratyn-Schaus Y. Pasqualini F. S. Yuan H. McCain M. L. Ye G. J. C. Sheehy S. P. (2016). Coupling primary and stem cell–derived cardiomyocytes in an *in vitro* model of cardiac cell therapy. J. Cell Biol. 212, 389–397. 10.1083/jcb.201508026 26858266 PMC4754718

[B4] Arshi A. Nakashima Y. Nakano H. Eaimkhong S. Evseenko D. Reed J. (2013). Rigid microenvironments promote cardiac differentiation of mouse and human embryonic stem cells. Sci. Technol. Adv. Mat. 14, 025003. 10.1088/1468-6996/14/2/025003 PMC3845966 24311969

[B5] Bedada F. B. Wheelwright M. Metzger J. M. (2016). Maturation status of sarcomere structure and function in human iPSC-derived cardiac myocytes. Biochimica Biophysica Acta Bba - Mol Cell Res 1863, 1829–1838. 10.1016/j.bbamcr.2015.11.005 PMC4864165 26578113

[B6] Bers D. M. (2002). Cardiac excitation–contraction coupling. Nature 415, 198–205. 10.1038/415198a 11805843

[B7] Block T. Creech J. Rocha A. M. Marinkovic M. Ponce-Balbuena D. Jiménez-Vázquez E. N. (2020). Human perinatal stem cell derived extracellular matrix enables rapid maturation of hiPSC-CM structural and functional phenotypes. Sci. Rep-uk 10, 19071. 10.1038/s41598-020-76052-y PMC7643060 33149250

[B8] Chirico N. Kessler E. L. Maas R. G. C. Fang J. Qin J. Dokter I. (2022). Small molecule-mediated rapid maturation of human induced pluripotent stem cell-derived cardiomyocytes. Stem Cell Res. Ther. 13, 531. 10.1186/s13287-022-03209-z 36575473 PMC9795728

[B9] Christoffels V. M. Smits G. J. Kispert A. Moorman A. F. M. (2010). Development of the pacemaker tissues of the heart. Circ. Res. 106, 240–254. 10.1161/circresaha.109.205419 20133910

[B10] Chun Y. W. Balikov D. A. Feaster T. K. Williams C. H. Sheng C. C. Lee J.-B. (2015). Combinatorial polymer matrices enhance *in vitro* maturation of human induced pluripotent stem cell-derived cardiomyocytes. Biomaterials 67, 52–64. 10.1016/j.biomaterials.2015.07.004 26204225 PMC4550551

[B11] Correia C. Koshkin A. Duarte P. Hu D. Teixeira A. Domian I. (2017). Distinct carbon sources affect structural and functional maturation of cardiomyocytes derived from human pluripotent stem cells. Sci. Rep-uk 7, 8590. 10.1038/s41598-017-08713-4 PMC5561128 28819274

[B12] Crestani T. Steichen C. Neri E. Rodrigues M. Fonseca-Alaniz M. H. Ormrod B. (2020). Electrical stimulation applied during differentiation drives the hiPSC-CMs towards a mature cardiac conduction-like cells. Biochem Bioph Res. Co. 533, 376–382. 10.1016/j.bbrc.2020.09.021 32962862

[B13] Cui C. Wang J. Qian D. Huang J. Lin J. Kingshott P. (2019). Binary colloidal crystals drive spheroid formation and accelerate maturation of human-induced pluripotent stem cell-derived cardiomyocytes. Acs Appl. Mater Inter 11, 3679–3689. 10.1021/acsami.8b17090 30614683

[B14] Dai D.-F. Danoviz M. E. Wiczer B. Laflamme M. A. Tian R. (2017). Mitochondrial maturation in human pluripotent stem cell derived cardiomyocytes. Stem Cells Int. 2017, 1–10. 10.1155/2017/5153625 PMC5380852 28421116

[B15] Dai W. Field L. J. Rubart M. Reuter S. Hale S. L. Zweigerdt R. (2007). Survival and maturation of human embryonic stem cell-derived cardiomyocytes in rat hearts. J. Mol. Cell Cardiol. 43, 504–516. 10.1016/j.yjmcc.2007.07.001 17707399 PMC2796607

[B16] Dhahri W. Valdman T. S. Wilkinson D. Pereira E. Ceylan E. Andharia N. (2022). *In vitro* matured human pluripotent stem cell–derived cardiomyocytes form grafts with enhanced structure and function in injured hearts. Circulation 145, 1412–1426. 10.1161/circulationaha.121.053563 35089805

[B17] Dinkova-Kostova A. T. Abramov A. Y. (2015). The emerging role of Nrf2 in mitochondrial function. Free Radic. Bio Med. 88, 179–188. 10.1016/j.freeradbiomed.2015.04.036 25975984 PMC4726722

[B18] Dorn G. W. Vega R. B. Kelly D. P. (2015). Mitochondrial biogenesis and dynamics in the developing and diseased heart. Gene Dev. 29, 1981–1991. 10.1101/gad.269894.115 26443844 PMC4604339

[B19] Dubois N. C. Craft A. M. Sharma P. Elliott D. A. Stanley E. G. Elefanty A. G. (2011). SIRPA is a specific cell-surface marker for isolating cardiomyocytes derived from human pluripotent stem cells. Nat. Biotechnol. 29, 1011–1018. 10.1038/nbt.2005 22020386 PMC4949030

[B20] Dufour C. R. Wilson B. J. Huss J. M. Kelly D. P. Alaynick W. A. Downes M. (2007). Genome-wide orchestration of cardiac functions by the orphan nuclear receptors ERRα and γ. Cell Metab. 5, 345–356. 10.1016/j.cmet.2007.03.007 17488637

[B21] Dunn K. K. Reichardt I. M. Simmons A. D. Jin G. Floy M. E. Hoon K. M. (2019). Coculture of endothelial cells with human pluripotent stem cell-derived cardiac progenitors reveals a differentiation stage‐specific enhancement of cardiomyocyte maturation. Biotechnol. J. 14, 1800725. 10.1002/biot.201800725 PMC6849481 30927511

[B22] Efraim Y. Sarig H. Anavy N. C. Sarig U. Berardinis E. Chaw S-Y. (2017). Biohybrid cardiac ECM-based hydrogels improve long term cardiac function post myocardial infarction. Acta Biomater. 50, 220–233. 10.1016/j.actbio.2016.12.015 27956366

[B23] Emanuelli G. Zoccarato A. Reumiller C. M. Papadopoulos A. Chong M. Rebs S. (2022). A roadmap for the characterization of energy metabolism in human cardiomyocytes derived from induced pluripotent stem cells. J. Mol. Cell Cardiol. 164, 136–147. 10.1016/j.yjmcc.2021.12.001 34923199

[B24] Eng G. Lee B. W. Protas L. Gagliardi M. Brown K. Kass R. S. (2016). Autonomous beating rate adaptation in human stem cell-derived cardiomyocytes. Nat. Commun. 7, 10312. 10.1038/ncomms10312 26785135 PMC4735644

[B25] Feaster T. K. Cadar A. G. Wang L. Williams C. H. Chun Y. W. Hempel J. E. (2015). Matrigel mattress. Circ. Res. 117, 995–1000. 10.1161/circresaha.115.307580 26429802 PMC4670592

[B26] Ferrantini C. Crocini C. Coppini R. Vanzi F. Tesi C. Cerbai E. (2013). The transverse-axial tubular system of cardiomyocytes. Cell Mol. Life Sci. 70, 4695–4710. 10.1007/s00018-013-1410-5 23846763 PMC11113601

[B27] Feyen D. A. M. McKeithan W. L. Bruyneel A. A. N. Spiering S. Hörmann L. Ulmer B. (2020). Metabolic maturation media improve physiological function of human iPSC-derived cardiomyocytes. Cell Rep. 32, 107925. 10.1016/j.celrep.2020.107925 32697997 PMC7437654

[B28] Fong A. H. Romero-López M. Heylman C. M. Keating M. Tran D. Sobrino A. (2016). Three-dimensional adult cardiac extracellular matrix promotes maturation of human induced pluripotent stem cell-derived cardiomyocytes. Tissue Eng. Pt A 22, 1016–1025. 10.1089/ten.tea.2016.0027 PMC4991595 27392582

[B29] Funakoshi S. Fernandes I. Mastikhina O. Wilkinson D. Tran T. Dhahri W. (2021). Generation of mature compact ventricular cardiomyocytes from human pluripotent stem cells. Nat. Commun. 12, 3155. 10.1038/s41467-021-23329-z 34039977 PMC8155185

[B30] Garay B. I. Givens S. Abreu P. Liu M. Yücel D. Baik J. (2022). Dual inhibition of MAPK and PI3K/AKT pathways enhances maturation of human iPSC-derived cardiomyocytes. Stem Cell Rep. 17, 2005–2022. 10.1016/j.stemcr.2022.07.003 PMC9481895 35931076

[B31] Gaspar J. A. Doss M. X. Hengstler J. G. Cadenas C. Hescheler J. Sachinidis A. (2014). Unique metabolic features of stem cells, cardiomyocytes, and their progenitors. Circ. Res. 114, 1346–1360. 10.1161/circresaha.113.302021 24723659

[B32] Gentillon C. Li D. Duan M. Yu W.-M. Preininger M. K. Jha R. (2019). Targeting HIF-1α in combination with PPARα activation and postnatal factors promotes the metabolic maturation of human induced pluripotent stem cell-derived cardiomyocytes. J. Mol. Cell Cardiol. 132, 120–135. 10.1016/j.yjmcc.2019.05.003 31082397 PMC6683286

[B33] Gherghiceanu M. Barad L. Novak A. Reiter I. Itskovitz-Eldor J. Binah O. (2011). Cardiomyocytes derived from human embryonic and induced pluripotent stem cells: Comparative ultrastructure. J. Cell Mol. Med. 15, 2539–2551. 10.1111/j.1582-4934.2011.01417.x 21883888 PMC3822963

[B34] Giacomelli E. Meraviglia V. Campostrini G. Cochrane A. Cao X. Helden R. W. J. (2020). Human-iPSC-Derived cardiac stromal cells enhance maturation in 3D cardiac microtissues and reveal non-cardiomyocyte contributions to heart disease. Cell Stem Cell 26, 862–879. e11. 10.1016/j.stem.2020.05.004 32459996 PMC7284308

[B35] Goldfracht I. Efraim Y. Shinnawi R. Kovalev E. Huber I. Gepstein A. (2019). Engineered heart tissue models from hiPSC-derived cardiomyocytes and cardiac ECM for disease modeling and drug testing applications. Acta Biomater. 92, 145–159. 10.1016/j.actbio.2019.05.016 31075518

[B36] Guo Y. Pu W. T. (2020). Cardiomyocyte maturation. Circ. Res. 126, 1086–1106. 10.1161/circresaha.119.315862 32271675 PMC7199445

[B37] Hampe N. Jonas T. Wolters B. Hersch N. Hoffmann B. Merkel R. (2014). Defined 2-D microtissues on soft elastomeric silicone rubber using lift-off epoxy-membranes for biomechanical analyses. Soft Matter 10, 2431–2443. 10.1039/c3sm53123f 24623394

[B38] Hazeltine L. B. Simmons C. S. Salick M. R. Lian X. Badur M. G. Han W. (2012). Effects of substrate mechanics on contractility of cardiomyocytes generated from human pluripotent stem cells. Int. J. Cell Biol. 2012, 1–13. 10.1155/2012/508294 PMC3357596 22649451

[B39] Herron T. J. Rocha A. M. D. Campbell K. F. Ponce-Balbuena D. Willis B. C. Guerrero-Serna G. (2018). Extracellular matrix–mediated maturation of human pluripotent stem cell–derived cardiac monolayer structure and electrophysiological function. Circ. Arrhythmia Electrophysiol. 9, e003638. 10.1161/circep.113.003638 PMC4833010 27069088

[B40] Hirt M. N. Boeddinghaus J. Mitchell A. Schaaf S. Börnchen C. Müller C. (2014). Functional improvement and maturation of rat and human engineered heart tissue by chronic electrical stimulation. J. Mol. Cell Cardiol. 74, 151–161. 10.1016/j.yjmcc.2014.05.009 24852842

[B41] Hoang P. Wang J. Conklin B. R. Healy K. E. Ma Z. (2018). Generation of spatial-patterned early-developing cardiac organoids using human pluripotent stem cells. Nat. Protoc. 13, 723–737. 10.1038/nprot.2018.006 29543795 PMC6287283

[B42] Hochman-Mendez C. Campos D. B. P. Pinto R. S. Mendes B. J. Rocha G. M. Monnerat G. (2020). Tissue-engineered human embryonic stem cell-containing cardiac patches: Evaluating recellularization of decellularized matrix. J. Tissue Eng. 11, 204173142092148. 10.1177/2041731420921482 PMC7375712 32742631

[B43] Horikoshi Y. Yan Y. Terashvili M. Wells C. Horikoshi H. Fujita S. (2019). Fatty acid-treated induced pluripotent stem cell-derived human cardiomyocytes exhibit adult cardiomyocyte-like energy metabolism phenotypes. Cells 8, 1095. 10.3390/cells8091095 31533262 PMC6769886

[B44] Hu D. Linders A. Yamak A. Correia C. Kijlstra J. D. Garakani A. (2018). Metabolic maturation of human pluripotent stem cell-derived cardiomyocytes by inhibition of HIF1α and LDHA. Circ. Res. 123, 1066–1079. 10.1161/circresaha.118.313249 30355156 PMC6208155

[B45] Huang C. Y. Maia-Joca R. P. M. Ong C. S. Wilson I. DiSilvestre D. Tomaselli G. F. (2020). Enhancement of human iPSC-derived cardiomyocyte maturation by chemical conditioning in a 3D environment. J. Mol. Cell Cardiol. 138, 1–11. 10.1016/j.yjmcc.2019.10.001 31655038

[B46] Huang N. F. Li S. (2011). Regulation of the matrix microenvironment for stem cell engineering and regenerative medicine. Ann. Biomed. Eng. 39, 1201–1214. 10.1007/s10439-011-0297-2 21424849 PMC3568678

[B47] Huebsch N. Charrez B. Neiman G. Siemons B. Boggess S. C. Wall S. (2022). Metabolically driven maturation of human-induced-pluripotent-stem-cell-derived cardiac microtissues on microfluidic chips. Nat. Biomed. Eng. 6, 372–388. 10.1038/s41551-022-00884-4 35478228 PMC10344596

[B48] Huethorst E. Hortigon M. Zamora-Rodriguez V. Reynolds P. M. Burton F. Smith G. (2016). Enhanced human-induced pluripotent stem cell derived cardiomyocyte maturation using a dual microgradient substrate. Acs Biomater. Sci. Eng. 2, 2231–2239. 10.1021/acsbiomaterials.6b00426 27990488 PMC5155309

[B49] Kadota S. Pabon L. Reinecke H. Murry C. E. (2017). *In vivo* maturation of human induced pluripotent stem cell-derived cardiomyocytes in neonatal and adult rat hearts. Stem Cell Rep. 8, 278–289. 10.1016/j.stemcr.2016.10.009 PMC5311430 28065644

[B50] Kahn-Krell A. Pretorius D. Guragain B. Lou X. Wei Y. Zhang J. (2022). A three-dimensional culture system for generating cardiac spheroids composed of cardiomyocytes, endothelial cells, smooth-muscle cells, and cardiac fibroblasts derived from human induced-pluripotent stem cells. Front. Bioeng. Biotechnol. 10, 908848. 10.3389/fbioe.2022.908848 35957645 PMC9361017

[B51] Kamakura T. Makiyama T. Sasaki K. Yoshida Y. Wuriyanghai Y. Chen J. (2013). Ultrastructural maturation of human-induced pluripotent stem cell-derived cardiomyocytes in a long-term culture. Circ. J. 77, 1307–1314. 10.1253/circj.cj-12-0987 23400258

[B52] Karbassi E. Fenix A. Marchiano S. Muraoka N. Nakamura K. Yang X. (2020). Cardiomyocyte maturation: Advances in knowledge and implications for regenerative medicine. Nat. Rev. Cardiol. 17, 341–359. 10.1038/s41569-019-0331-x 32015528 PMC7239749

[B53] Kattman S. J. Witty A. D. Gagliardi M. Dubois N. C. Niapour M. Hotta A. (2011). Stage-specific optimization of activin/nodal and BMP signaling promotes cardiac differentiation of mouse and human pluripotent stem cell lines. Cell Stem Cell 8, 228–240. 10.1016/j.stem.2010.12.008 21295278

[B54] Kawai Y. Tohyama S. Arai K. Tamura T. Soma Y. Fukuda K. (2022). Scaffold-free tubular engineered heart tissue from human induced pluripotent stem cells using bio-3D printing technology *in vivo* . Front. Cardiovasc Med. 8, 806215. 10.3389/fcvm.2021.806215 35127867 PMC8811174

[B55] Kensah G. Lara A. R. Dahlmann J. Zweigerdt R. Schwanke K. Hegermann J. (2013). Murine and human pluripotent stem cell-derived cardiac bodies form contractile myocardial tissue *in vitro* . Eur. Heart J. 34, 1134–1146. 10.1093/eurheartj/ehs349 23103664

[B56] Khan M. Xu Y. Hua S. Johnson J. Belevych A. Janssen P. M. L. (2015). Evaluation of changes in morphology and function of human induced pluripotent stem cell derived cardiomyocytes (HiPSC-CMs) cultured on an aligned-nanofiber cardiac patch. Plos One 10, e0126338. 10.1371/journal.pone.0126338 25993466 PMC4437999

[B57] Kim C. Majdi M. Xia P. Wei K. A. Talantova M. Spiering S. (2010). Non-cardiomyocytes influence the electrophysiological maturation of human embryonic stem cell-derived cardiomyocytes during differentiation. Stem Cells Dev. 19, 783–795. 10.1089/scd.2009.0349 20001453 PMC3135229

[B58] Kim Y. S. Yoon J. W. Kim D. Choi S. Kim H. K. Youm J. B. (2022). Tomatidine-stimulated maturation of human embryonic stem cell-derived cardiomyocytes for modeling mitochondrial dysfunction. Exp. Mol. Med. 54, 493–502. 10.1038/s12276-022-00746-8 35379934 PMC9076832

[B59] Knight W. E. Cao Y. Lin Y.-H. Chi C. Bai B. Sparagna G. C. (2021). Maturation of pluripotent stem cell-derived cardiomyocytes enables modeling of human hypertrophic cardiomyopathy. Stem Cell Rep. 16, 519–533. 10.1016/j.stemcr.2021.01.018 PMC7940251 33636116

[B60] Kolanowski T. J. Busek M. Schubert M. Dmitrieva A. Binnewerg B. Pöche J. (2020). Enhanced structural maturation of human induced pluripotent stem cell-derived cardiomyocytes under a controlled microenvironment in a microfluidic system. Acta Biomater. 102, 273–286. 10.1016/j.actbio.2019.11.044 31778832

[B61] Kuppusamy K. T. Jones D. C. Sperber H. Madan A. Fischer K. A. Rodriguez M. L. (2015). Let-7 family of microRNA is required for maturation and adult-like metabolism in stem cell-derived cardiomyocytes. Proc. Natl. Acad. Sci. 112, E2785–E2794. 10.1073/pnas.1424042112 25964336 PMC4450404

[B62] Laake L. W. Passier R. Monshouwer-Kloots J. Verkleij A. J. Lips D. J. Freund C. (2007). Human embryonic stem cell-derived cardiomyocytes survive and mature in the mouse heart and transiently improve function after myocardial infarction. Stem Cell Res. 1, 9–24. 10.1016/j.scr.2007.06.001 19383383

[B63] Lee D. S. Chen J.-H. Lundy D. J. Liu C.-H. Hwang S.-M. Pabon L. (2015). Defined MicroRNAs induce aspects of maturation in mouse and human embryonic-stem-cell-derived cardiomyocytes. Cell Rep. 12, 1960–1967. 10.1016/j.celrep.2015.08.042 26365191

[B64] Li F. Wang X. Capasso J. M. Gerdes A. M. (1996). Rapid transition of cardiac myocytes from hyperplasia to hypertrophy during postnatal development. J. Mol. Cell Cardiol. 28, 1737–1746. 10.1006/jmcc.1996.0163 8877783

[B65] Li M. Iismaa S. E. Naqvi N. Nicks A. Husain A. Graham R. M. (2014). Thyroid hormone action in postnatal heart development. Stem Cell Res. 13, 582–591. 10.1016/j.scr.2014.07.001 25087894

[B66] Lian X. Hsiao C. Wilson G. Zhu K. Hazeltine L. B. Azarin S. M. (2012). Robust cardiomyocyte differentiation from human pluripotent stem cells via temporal modulation of canonical Wnt signaling. Proc. Natl. Acad. Sci. 109, E1848–E1857. 10.1073/pnas.1200250109 22645348 PMC3390875

[B67] Lin B. Lin X. Stachel M. Wang E. Luo Y. Lader J. (2017). Culture in glucose-depleted medium supplemented with fatty acid and 3,3′,5-Triiodo-l-Thyronine facilitates purification and maturation of human pluripotent stem cell-derived cardiomyocytes. Front. Endocrinol. 8, 253. 10.3389/fendo.2017.00253 PMC5641374 29067001

[B68] Liu A. Tang M. Xi J. Gao L. Zheng Y. Luo H. (2010). Functional characterization of inward rectifier potassium ion channel in murine fetal ventricular cardiomyocytes. Cell Physiol. Biochem 26, 413–420. 10.1159/000320565 20798526

[B69] Liu Y.-W. Chen B. Yang X. Fugate J. A. Kalucki F. A. Futakuchi-Tsuchida A. (2018). Human embryonic stem cell–derived cardiomyocytes restore function in infarcted hearts of non-human primates. Nat. Biotechnol. 36, 597–605. 10.1038/nbt.4162 29969440 PMC6329375

[B70] Liu Y. Bai H. Guo F. Thai P. N. Luo X. Zhang P. (2020). PGC-1α activator ZLN005 promotes maturation of cardiomyocytes derived from human embryonic stem cells. Aging Albany Ny 12, 7411–7430. 10.18632/aging.103088 32343674 PMC7202542

[B71] Lopez C. A. Al-Siddiqi H. H. A. A. Purnama U. Iftekhar S. Bruyneel A. A. N. Kerr M. (2021). Physiological and pharmacological stimulation for *in vitro* maturation of substrate metabolism in human induced pluripotent stem cell-derived cardiomyocytes. Sci. Rep-uk 11, 7802. 10.1038/s41598-021-87186-y PMC8032667 33833285

[B72] Lu K. Seidel T. Cao-Ehlker X. Dorn T. Batcha A. M. N. Schneider C. M. (2021). Progressive stretch enhances growth and maturation of 3D stem-cell-derived myocardium. Theranostics 11, 6138–6153. 10.7150/thno.54999 33995650 PMC8120210

[B73] Lundy S. D. Zhu W.-Z. Regnier M. Laflamme M. A. (2013). Structural and functional maturation of cardiomyocytes derived from human pluripotent stem cells. Stem Cells Dev. 22, 1991–2002. 10.1089/scd.2012.0490 23461462 PMC3699903

[B74] Ma J. Guo L. Fiene S. J. Anson B. D. Thomson J. A. Kamp T. J. (2011). High purity human-induced pluripotent stem cell-derived cardiomyocytes: Electrophysiological properties of action potentials and ionic currents. Am. J. Physiol-heart C 301, H2006–H2017. 10.1152/ajpheart.00694.2011 PMC4116414 21890694

[B75] Ma Z. Wang J. Loskill P. Huebsch N. Koo S. Svedlund F. L. (2015). Self-organizing human cardiac microchambers mediated by geometric confinement. Nat. Commun. 6, 7413. 10.1038/ncomms8413 26172574 PMC4503387

[B76] Makinde A.-O. Kantor P. F. Lopaschuk G. D. (1998). Maturation of fatty acid and carbohydrate metabolism in the newborn heart. Mol. Cell Biochem 188, 49–56. 10.1023/a:1006860104840 9823010

[B77] Maleckar M. M. Edwards A. G. Louch W. E. Lines G. T. (2017). Studying dyadic structure–function relationships: A review of current modeling approaches and new insights into Ca2+ (mis)handling. Clin. Med. Insights Cardiol. 11, 117954681769860. 10.1177/1179546817698602 PMC5392018 28469494

[B78] Mesquita F. C. P. Morrissey J. Monnerat G. Domont G. B. Nogueira F. C. S. Hochman-Mendez C. (2021). Decellularized extracellular matrix powder accelerates metabolic maturation at early stages of cardiac differentiation in human induced pluripotent stem cell-derived cardiomyocytes. Cells Tissues Organs 212, 32–44. 10.1159/000521580 34933302

[B79] Mihic A. Li J. Miyagi Y. Gagliardi M. Li S.-H. Zu J. (2014). The effect of cyclic stretch on maturation and 3D tissue formation of human embryonic stem cell-derived cardiomyocytes. Biomaterials 35, 2798–2808. 10.1016/j.biomaterials.2013.12.052 24424206

[B80] Mousavi A. Stefanek E. Jafari A. Ajji Z. Naghieh S. Akbari M. (2022). Tissue-engineered heart chambers as a platform technology for drug discovery and disease modeling. Biomater. Adv. 138, 212916. 10.1016/j.bioadv.2022.212916 35913255

[B81] Muñoz J. J. A. M. Dariolli R. Silva C. M. Neri E. A. Valadão I. C. Turaça L. T. (2022). Time-regulated transcripts with the potential to modulate human pluripotent stem cell-derived cardiomyocyte differentiation. Stem Cell Res. Ther. 13, 437. 10.1186/s13287-022-03138-x 36056380 PMC9438174

[B82] Murphy S. A. Miyamoto M. Kervadec A. Kannan S. Tampakakis E. Kambhampati S. (2021). PGC1/PPAR drive cardiomyocyte maturation at single cell level via YAP1 and SF3B2. Nat. Commun. 12, 1648. 10.1038/s41467-021-21957-z 33712605 PMC7955035

[B83] Nakano H. Minami I. Braas D. Pappoe H. Wu X. Sagadevan A. (2017). Glucose inhibits cardiac muscle maturation through nucleotide biosynthesis. Elife 6, e29330. 10.7554/elife.29330 29231167 PMC5726851

[B84] Ni X. Xu K. Zhao Y. Li J. Wang L. Yu F. (2021). Single-cell analysis reveals the purification and maturation effects of glucose starvation in hiPSC-CMs. Biochem Bioph Res. Co. 534, 367–373. 10.1016/j.bbrc.2020.11.076 33279112

[B85] Nunes S. S. Miklas J. W. Liu J. Aschar-Sobbi R. Xiao Y. Zhang B. (2013). Biowire: A platform for maturation of human pluripotent stem cell–derived cardiomyocytes. Nat. Methods 10, 781–787. 10.1038/nmeth.2524 23793239 PMC4071061

[B87] Parikh S. S. Blackwell D. J. Gomez-Hurtado N. Frisk M. Wang L. Kim K. (2017). Thyroid and glucocorticoid hormones promote functional T-tubule development in human-induced pluripotent stem cell–derived cardiomyocytes. Circ. Res. 121, 1323–1330. 10.1161/circresaha.117.311920 28974554 PMC5722667

[B88] Pinto A. R. Ilinykh A. Ivey M. J. Kuwabara J. T. D’Antoni M. L. Debuque R. (2016). Revisiting cardiac cellular composition. Circ. Res. 118, 400–409. 10.1161/circresaha.115.307778 26635390 PMC4744092

[B89] Ramachandra C. J. A. Mehta A. Wong P. Ja K. P. M. M. Fritsche-Danielson R. Bhat R. V. (2018). Fatty acid metabolism driven mitochondrial bioenergetics promotes advanced developmental phenotypes in human induced pluripotent stem cell derived cardiomyocytes. Int. J. Cardiol. 272, 288–297. 10.1016/j.ijcard.2018.08.069 30177232

[B90] Ramirez-Calderon G. Colombo G. Hernandez-Bautista C. A. Astro V. Adamo A. (2022). Heart in a dish: From traditional 2D differentiation protocols to cardiac organoids. Front. Cell Dev. Biol. 10, 855966. 10.3389/fcell.2022.855966 35252213 PMC8893312

[B91] Rao C. Prodromakis T. Kolker L. Chaudhry U. A. R. Trantidou T. Sridhar A. (2013). The effect of microgrooved culture substrates on calcium cycling of cardiac myocytes derived from human induced pluripotent stem cells. Biomaterials 34, 2399–2411. 10.1016/j.biomaterials.2012.11.055 23261219 PMC3605579

[B92] Reilly L. Munawar S. Zhang J. Crone W. C. Eckhardt L. L. (2022). Challenges and innovation: Disease modeling using human-induced pluripotent stem cell-derived cardiomyocytes. Front. Cardiovasc Med. 9, 966094. 10.3389/fcvm.2022.966094 36035948 PMC9411865

[B94] Ribeiro A. J. S. Ang Y.-S. Fu J.-D. Rivas R. N. Mohamed T. M. A. Higgs G. C. (2015). Contractility of single cardiomyocytes differentiated from pluripotent stem cells depends on physiological shape and substrate stiffness. Proc. Natl. Acad. Sci. 112, 12705–12710. 10.1073/pnas.1508073112 26417073 PMC4611612

[B95] Rienks M. Papageorgiou A.-P. Frangogiannis N. G. Heymans S. (2014). Myocardial extracellular matrix. Circ. Res. 114, 872–888. 10.1161/circresaha.114.302533 24577967

[B96] Rodriguez M. L. Beussman K. M. Chun K. S. Walzer M. S. Yang X. Murry C. E. (2019). Substrate stiffness, cell anisotropy, and cell–cell contact contribute to enhanced structural and calcium handling properties of human embryonic stem cell-derived cardiomyocytes. Acs Biomater. Sci. Eng. 5, 3876–3888. 10.1021/acsbiomaterials.8b01256 33438427

[B97] Rog-Zielinska E. A. Craig M.-A. Manning J. R. Richardson R. V. Gowans G. J. Dunbar D. R. (2015). Glucocorticoids promote structural and functional maturation of foetal cardiomyocytes: A role for PGC-1α. Cell Death Differ. 22, 1106–1116. 10.1038/cdd.2014.181 25361084 PMC4572859

[B98] Rog-Zielinska E. A. Thomson A. Kenyon C. J. Brownstein D. G. Moran C. M. Szumska D. (2013). Glucocorticoid receptor is required for foetal heart maturation. Hum. Mol. Genet. 22, 3269–3282. 10.1093/hmg/ddt182 23595884

[B99] Ruan J. Tulloch N. L. Saiget M. Paige S. L. Razumova M. V. Regnier M. (2015). Mechanical stress promotes maturation of human myocardium from pluripotent stem cell‐derived progenitors. Stem Cells 33, 2148–2157. 10.1002/stem.2036 25865043 PMC4478130

[B100] Sachs P. C. Mollica P. A. Bruno R. D. (2017). Tissue specific microenvironments: A key tool for tissue engineering and regenerative medicine. J. Biol. Eng. 11, 34. 10.1186/s13036-017-0077-0 29177006 PMC5688702

[B101] Sakamoto T. Matsuura T. R. Wan S. Ryba D. M. Kim J. Won K. J. (2020). A critical role for estrogen-related receptor signaling in cardiac maturation. Circ. Res. 126, 1685–1702. 10.1161/circresaha.119.316100 32212902 PMC7274895

[B102] Salick M. R. Napiwocki B. N. Sha J. Knight G. T. Chindhy S. A. Kamp T. J. (2014). Micropattern width dependent sarcomere development in human ESC-derived cardiomyocytes. Biomaterials 35, 4454–4464. 10.1016/j.biomaterials.2014.02.001 24582552 PMC4026015

[B103] Schaefer J. A. Guzman P. A. Riemenschneider S. B. Kamp T. J. Tranquillo R. T. (2018). A cardiac patch from aligned microvessel and cardiomyocyte patches. J. Tissue Eng. Regen. M. 12, 546–556. 10.1002/term.2568 28875579 PMC5814344

[B104] Shadrin I. Y. Allen B. W. Qian Y. Jackman C. P. Carlson A. L. Juhas M. E. (2017). Cardiopatch platform enables maturation and scale-up of human pluripotent stem cell-derived engineered heart tissues. Nat. Commun. 8, 1825. 10.1038/s41467-017-01946-x 29184059 PMC5705709

[B105] Sharma A. Wu J. C. Wu S. M. (2013). Induced pluripotent stem cell-derived cardiomyocytes for cardiovascular disease modeling and drug screening. Stem Cell Res. Ther. 4, 150. 10.1186/scrt380 24476344 PMC4056681

[B106] Shen N. Knopf A. Westendorf C. Kraushaar U. Riedl J. Bauer H. (2017). Steps toward maturation of embryonic stem cell-derived cardiomyocytes by defined physical signals. Stem Cell Rep. 9, 122–135. 10.1016/j.stemcr.2017.04.021 PMC5511039 28528699

[B107] Shen S. Sewanan L. R. Shao S. Halder S. S. Stankey P. Li X. (2022). Physiological calcium combined with electrical pacing accelerates maturation of human engineered heart tissue. Stem Cell Rep. 17, 2037–2049. 10.1016/j.stemcr.2022.07.006 PMC9481907 35931080

[B108] Silbernagel N. Körner A. Balitzki J. Jaggy M. Bertels S. Richter B. (2020). Shaping the heart: Structural and functional maturation of iPSC-cardiomyocytes in 3D-micro-scaffolds. Biomaterials 227, 119551. 10.1016/j.biomaterials.2019.119551 31670034

[B109] Sim C. B. Phipson B. Ziemann M. Rafehi H. Mills R. J. Watt K. I. (2021). Sex-specific control of human heart maturation by the progesterone receptor. Circulation 143, 1614–1628. 10.1161/circulationaha.120.051921 33682422 PMC8055196

[B110] Song M. Jang Y. Kim S.-J. Park Y. (2022). Cyclic stretching induces maturation of human-induced pluripotent stem cell-derived cardiomyocytes through nuclear-mechanotransduction. Tissue Eng. Regen. Med. 19, 781–792. 10.1007/s13770-021-00427-z 35258794 PMC9294081

[B111] Strimaityte D. Tu C. Yanez A. Itzhaki I. Wu H. Wu J. C. (2022). Contractility and calcium transient maturation in the human iPSC-derived cardiac microfibers. Acs Appl. Mater Inter 14, 35376–35388. 10.1021/acsami.2c07326 PMC9780031 35901275

[B112] Sun X. Nunes S. S. (2017). Maturation of human stem cell-derived cardiomyocytes in biowires using electrical stimulation. J. Vis. Exp. Jove 123, 55373. 10.3791/55373 PMC5607897 28518082

[B113] Sun X. Wu J. Qiang B. Romagnuolo R. Gagliardi M. Keller G. (2020). Transplanted microvessels improve pluripotent stem cell–derived cardiomyocyte engraftment and cardiac function after infarction in rats. Sci. Transl. Med. 12, eaax2992. 10.1126/scitranslmed.aax2992 32967972

[B114] Takahashi K. Tanabe K. Ohnuki M. Narita M. Ichisaka T. Tomoda K. (2007). Induction of pluripotent stem cells from adult human fibroblasts by defined factors. Cell 131, 861–872. 10.1016/j.cell.2007.11.019 18035408

[B115] Thavandiran N. Dubois N. Mikryukov A. Massé S. Beca B. Simmons C. A. (2013). Design and formulation of functional pluripotent stem cell-derived cardiac microtissues. Proc. Natl. Acad. Sci. 110, E4698–E4707. 10.1073/pnas.1311120110 24255110 PMC3856835

[B116] Thomson J. A. Itskovitz-Eldor J. Shapiro S. S. Waknitz M. A. Swiergiel J. J. Marshall V. S. (1998). Embryonic stem cell lines derived from human blastocysts. Science 282, 1145–1147. 10.1126/science.282.5391.1145 9804556

[B117] Tohyama S. Hattori F. Sano M. Hishiki T. Nagahata Y. Matsuura T. (2013). Distinct metabolic flow enables large-scale purification of mouse and human pluripotent stem cell-derived cardiomyocytes. Cell Stem Cell 12, 127–137. 10.1016/j.stem.2012.09.013 23168164

[B118] Tulloch N. L. Muskheli V. Razumova M. V. Korte F. S. Regnier M. Hauch K. D. (2011). Growth of engineered human myocardium with mechanical loading and vascular coculture. Circ. Res. 109, 47–59. 10.1161/circresaha.110.237206 21597009 PMC3140796

[B119] Turnbull I. C. Karakikes I. Serrao G. W. Backeris P. Lee J. Xie C. (2014). Advancing functional engineered cardiac tissues toward a preclinical model of human myocardium. Faseb J. 28, 644–654. 10.1096/fj.13-228007 24174427 PMC3898643

[B120] Ulmer B. M. Stoehr A. Schulze M. L. Patel S. Gucek M. Mannhardt I. (2018). Contractile work contributes to maturation of energy metabolism in hiPSC-derived cardiomyocytes. Stem Cell Rep. 10, 834–847. 10.1016/j.stemcr.2018.01.039 PMC5919410 29503093

[B121] Varzideh F. Pahlavan S. Ansari H. Halvaei M. Kostin S. Feiz M.-S. (2019). Human cardiomyocytes undergo enhanced maturation in embryonic stem cell-derived organoid transplants. Biomaterials 192, 537–550. 10.1016/j.biomaterials.2018.11.033 30529872

[B122] Veldhuizen J. Cutts J. Brafman D. A. Migrino R. Q. Nikkhah M. (2020). Engineering anisotropic human stem cell-derived three-dimensional cardiac tissue on-a-chip. Biomaterials 256, 120195. 10.1016/j.biomaterials.2020.120195 32623207

[B123] Vreeker A. Stuijvenberg L. V. Hund T. J. Mohler P. J. Nikkels P. G. J. Veen T. A. B. (2014). Assembly of the cardiac intercalated disk during pre- and postnatal development of the human heart. Plos One 9, e94722. 10.1371/journal.pone.0094722 24733085 PMC3986238

[B124] Wang K. Lin R.-Z. Hong X. Ng A. H. Lee C. N. Neumeyer J. (2020). Robust differentiation of human pluripotent stem cells into endothelial cells via temporal modulation of ETV2 with modified mRNA. Sci. Adv. 6, eaba7606. 10.1126/sciadv.aba7606 32832668 PMC7439318

[B125] Wang L. Wada Y. Ballan N. Schmeckpeper J. Huang J. Rau C. D. (2021). Triiodothyronine and dexamethasone alter potassium channel expression and promote electrophysiological maturation of human-induced pluripotent stem cell-derived cardiomyocytes. J. Mol. Cell Cardiol. 161, 130–138. 10.1016/j.yjmcc.2021.08.005 34400182 PMC9809541

[B126] Wheelwright M. Win Z. Mikkila J. L. Amen K. Y. Alford P. W. Metzger J. M. (2018). Investigation of human iPSC-derived cardiac myocyte functional maturation by single cell traction force microscopy. Plos One 13, e0194909. 10.1371/journal.pone.0194909 29617427 PMC5884520

[B127] Wickramasinghe N. M. Sachs D. Shewale B. Gonzalez D. M. Dhanan-Krishnan P. Torre D. (2022). PPARdelta activation induces metabolic and contractile maturation of human pluripotent stem cell-derived cardiomyocytes. Cell Stem Cell 29, 559–576. e7. 10.1016/j.stem.2022.02.011 35325615 PMC11072853

[B128] Xiao X. Wang M. Qiu X. Ling W. Chu X. Huang Y. (2021). Construction of extracellular matrix-based 3D hydrogel and its effects on cardiomyocytes. Exp. Cell Res. 408, 112843. 10.1016/j.yexcr.2021.112843 34563515

[B129] Yang X. Rodriguez M. L. Leonard A. Sun L. Fischer K. A. Wang Y. (2019). Fatty acids enhance the maturation of cardiomyocytes derived from human pluripotent stem cells. Stem Cell Rep. 13, 657–668. 10.1016/j.stemcr.2019.08.013 PMC6829750 31564645

[B130] Yang X. Rodriguez M. Pabon L. Fischer K. A. Reinecke H. Regnier M. (2014). Tri-iodo-l-thyronine promotes the maturation of human cardiomyocytes-derived from induced pluripotent stem cells. J. Mol. Cell Cardiol. 72, 296–304. 10.1016/j.yjmcc.2014.04.005 24735830 PMC4041732

[B131] Ye L. Zhang X. Zhou Q. Tan B. Xu H. Yi Q. (2021). Activation of AMPK promotes maturation of cardiomyocytes derived from human induced pluripotent stem cells. Front. Cell Dev. Biol. 9, 644667. 10.3389/fcell.2021.644667 33768096 PMC7985185

[B132] Yoshida S. Miyagawa S. Fukushima S. Kawamura T. Kashiyama N. Ohashi F. (2018). Maturation of human induced pluripotent stem cell-derived cardiomyocytes by soluble factors from human mesenchymal stem cells. Mol. Ther. 26, 2681–2695. 10.1016/j.ymthe.2018.08.012 30217728 PMC6224789

[B133] Yu C. Ma X. Zhu W. Wang P. Miller K. L. Stupin J. (2019). Scanningless and continuous 3D bioprinting of human tissues with decellularized extracellular matrix. Biomaterials 194, 1–13. 10.1016/j.biomaterials.2018.12.009 30562651 PMC6339581

[B134] Yu J. Seldin M. M. Fu K. Li S. Lam L. Wang P. (2018). Topological arrangement of cardiac fibroblasts regulates cellular plasticity. Circ. Res. 123, 73–85. 10.1161/circresaha.118.312589 29691232 PMC6014922

[B135] Zhang D. Shadrin I. Y. Lam J. Xian H.-Q. Snodgrass H. R. Bursac N. (2013). Tissue-engineered cardiac patch for advanced functional maturation of human ESC-derived cardiomyocytes. Biomaterials 34, 5813–5820. 10.1016/j.biomaterials.2013.04.026 23642535 PMC3660435

[B136] Zhang W. Kong C. W. Tong M. H. Chooi W. H. Huang N. Li R. A. (2017). Maturation of human embryonic stem cell-derived cardiomyocytes (hESC-CMs) in 3D collagen matrix: Effects of niche cell supplementation and mechanical stimulation. Acta Biomater. 49, 204–217. 10.1016/j.actbio.2016.11.058 27890729

[B137] Zhang X. Ye L. Xu H. Zhou Q. Tan B. Yi Q. (2021). NRF2 is required for structural and metabolic maturation of human induced pluripotent stem cell-derived ardiomyocytes. Stem Cell Res. Ther. 12, 208. 10.1186/s13287-021-02264-2 33762018 PMC7992990

[B138] Zhao Y. Rafatian N. Wang E. Y. Feric N. T. Lai B. F. L. Knee-Walden E. J. (2020). Engineering microenvironment for human cardiac tissue assembly in heart-on-a-chip platform. Matrix Biol. 85, 189–204. 10.1016/j.matbio.2019.04.001 30981898 PMC6788963

